# del Nido versus St. Thomas’ blood cardioplegia in the young (DESTINY) trial: protocol for a multicentre randomised controlled trial in children undergoing cardiac surgery

**DOI:** 10.1136/bmjopen-2025-102029

**Published:** 2025-04-14

**Authors:** Nigel E Drury, Kelly Handley, Hugh Jarrett, Tina Griffin, Yongzhong Sun, Indie Bilkhoo, Alex Robertson, Carly Tooke, Barnaby R Scholefield, Warwick B Dunn, Martin Kostolny, Serban Stoica, Carin van Doorn, John V Pappachan, Timothy J Jones, Massimo Caputo

**Affiliations:** 1 Department of Cardiovascular Sciences, University of Birmingham, Birmingham, UK; 2 Department of Paediatric Cardiac Surgery, Birmingham Children’s Hospital, Birmingham, UK; 3 Birmingham Clinical Trials Unit, University of Birmingham, Birmingham, UK; 4 Department of Applied Health Sciences, University of Birmingham, Birmingham, UK; 5 Nottingham Clinical Trials Unit, University of Nottingham, Nottingham, UK; 6 Department of Paediatric Cardiac Surgery, Great Ormond Street Hospital for Children, London, UK; 7 Paediatric Intensive Care, Birmingham Children’s Hospital, Birmingham, UK; 8 Critical Care Medicine, Hospital for Sick Children, Toronto, Ontario, Canada; 9 Department of Paediatrics, University of Toronto, Toronto, Ontario, Canada; 10 Centre for Metabolomics Research, Biochemistry, Cell and Systems Biology, University of Liverpool, Liverpool, UK; 11 Department of Paediatric Cardiac Surgery, Bristol Royal Hospital for Children, Bristol, UK; 12 Bristol Medical School, University of Bristol, Bristol, UK; 13 Department of Congenital Cardiac Surgery, Leeds Children’s Hospital, Leeds, UK; 14 Paediatric Intensive Care, Southampton General Hospital, Southampton, UK; 15 Faculty of Medicine, University of Southampton, Southampton, UK; 16 NIHR Southampton Biomedical Research Centre, Southampton, UK; 17 Bristol Heart Institute, University of Bristol, Bristol, UK

**Keywords:** Paediatric cardiac surgery, Clinical Trial, Congenital heart disease

## Abstract

**Introduction:**

Myocardial protection against ischaemia–reperfusion injury is a key determinant of heart function and outcome following cardiac surgery in children. However, myocardial injury still occurs routinely following aortic cross-clamping, as demonstrated by the ubiquitous rise in circulating troponin. del Nido cardioplegia was designed to protect the immature myocardium and is widely used in the USA but has not previously been available in the UK, where St. Thomas’ blood cardioplegia is most common. The del Nido versus St. Thomas’ blood cardioplegia in the young (DESTINY) trial will evaluate whether one solution is better than the other at improving myocardial protection by reducing myocardial injury, shortening ischaemic time and improving clinical outcomes.

**Methods and analysis:**

The DESTINY trial is a multicentre, patient-blinded and assessor-blinded, parallel-group, individually randomised controlled trial recruiting up to 220 children undergoing surgery for congenital heart disease. Participants will be randomised in a 1:1 ratio to either del Nido cardioplegia or St. Thomas’ blood cardioplegia, with follow-up until 30 days following surgery. The primary outcome is area under the time–concentration curve for plasma high-sensitivity troponin I in the first 24 hours after aortic cross-clamp release. Secondary outcome measures include the incidence of low cardiac output syndrome and Vasoactive-Inotropic Score in the first 48 hours, total aortic cross-clamp time, duration of mechanical ventilation and lengths of stay in the paediatric intensive care unit and the hospital.

**Ethics and dissemination:**

The trial was approved by the West Midlands—Coventry and Warwickshire National Health Service Research Ethics Committee (21/WM/0149) on 30 June 2021. Findings will be disseminated to the academic community through peer-reviewed publications and presentation at national and international meetings. Parents will be informed of the results through a newsletter in conjunction with a national charity.

**Trial registration number:**

ISRCTN13638147; Pre-results.

**STRENGTHS AND LIMITATIONS OF THIS STUDY**

- This multicentre randomised controlled trial will compare two types of cardioplegia in children, recruiting across several paediatric cardiac surgical centres in the UK.
- The broad inclusion criteria, limited exclusions and pragmatic nature of the protocol will support the real-world nature of the trial and ensure the generalisability of its findings.
- Through the trial, we will make del Nido cardioplegia available in the UK for the first time, with an extended 12-month refrigerated shelf-life, thereby evaluating a product that is viable for ongoing use in the National Health Service.
- A potential limitation is the need for the operating surgeon, perfusionist, anaesthetist and theatre team to be unblinded to the allocation to protect patient safety, as cardioplegia delivery and dosing intervals necessarily differ between treatment groups, although patients and outcome assessors will be blinded.
- The effect of the intervention may be concealed if incision or resection of ventricular myocardium is performed, which may significantly increase troponin release above that associated with ischaemia–reperfusion.

## Introduction

### Myocardial protection with cardioplegia

 During most surgery for congenital heart disease, it is necessary to stop the heart, allowing access to a still and bloodless field to enable repair of intracardiac defects. Cardioplegia has been fundamental to arresting the heart and protecting against ischaemia–reperfusion injury during surgery for over 40 years, with approximately 3200 cardiac surgical operations with cardioplegic arrest performed in children in the UK and Ireland each year.^1^ While on cardiopulmonary bypass (CPB), a cross-clamp is placed across the proximal aorta and cardioplegia infused into the coronary arteries via the aortic root, leading to electromechanical arrest. This reduces myocardial oxygen uptake by 90%, and progressive hypothermia leads to a further stepwise reduction.^2^ However, myocardial injury still occurs routinely following aortic cross-clamping in children, as demonstrated by the ubiquitous release of troponin after surgery.^3 4^ Myocardial protection, therefore, is a key determinant of heart function and outcome following cardiac surgery, and improving organ protection during surgery was recently identified as the top national priority for research in children with congenital heart disease.^5^

Current paediatric cardioplegia techniques are primarily derived from adult practice or laboratory models; however, the immature myocardium has significant structural, physiological and metabolic differences from the adult heart, including sarcoplasmic reticulum development, mitochondrial density, substrate utilisation, calcium handling and antioxidant defences.^3^ It is less tolerant of ischaemia and more sensitive to calcium overload-mediated injury during reperfusion, particularly with hypoxaemia.^6 7^ Myocardial protection that is effective in adults therefore may not be optimal for young children,^8 9^ especially neonates and those with preoperative cyanosis.^10^ However, there are no late phase clinical trials of cardioplegia in children; most published trials are small, low quality, single-centre studies, with inconsistent endpoints and at risk of systematic bias.^11^ Of concern, these trials have recruited very few neonates, a high-risk group in whom the effects of cardioplegia are less well understood.^3^ Identifying the best cardioplegia for specific patient groups will enable the care of the child undergoing surgery to be individualised, potentially improve outcomes by reducing myocardial injury, morbidity and costs and may improve long-term cardiac function.^12^

Many types of cardioplegia solution are available, and there is wide variation in their use worldwide.^13^ In North America, del Nido cardioplegia is the most commonly used solution in children, with a recent survey finding its use by 78% of respondents from the Congenital Heart Surgeons’ Society.^14^ On the other hand, St. Thomas’ blood cardioplegia is used by most paediatric cardiac surgeons in most centres in the UK,^15^ where del Nido solution is not currently commercially available. There are significant theoretical and practical differences between these two autologous blood cardioplegia solutions; the usual interval between doses of del Nido is much longer (60–90 min)^16^ than for St. Thomas’ (20–30 min),^15^ such that in most cases, only a single dose is required. Eliminating or reducing the need to interrupt surgical flow to re-dose with cardioplegia improves the efficiency of the operation^17^ and may shorten the overall burden of ischaemia (cross-clamp time).^18^ Duration of aortic cross-clamp is an independent predictor of outcome after surgery,^19^ and therefore purely by reducing the duration of ischaemia, del Nido cardioplegia might be expected to reduce myocardial injury and potentially improve outcomes.

### Assessment of myocardial protection

Low cardiac output syndrome (LCOS) in the early postoperative period reflects the degree of myocardial injury and the need for inotropic support to maintain adequate systemic oxygen delivery. The presence of LCOS is a major determinant of outcome after heart surgery in children, and most deaths in the early postoperative period are attributed to a low cardiac output.^20 21^ However, there are no widely accepted methods for directly measuring cardiac output in young children, and therefore the identification of low cardiac output is dependent on the use of surrogate clinical signs such as need for inotropic support, arterial lactate concentration or omega.

Postoperative elevation of plasma troponin is a marker of myocardial injury and has been shown to strongly correlate with clinical outcomes including inotropic support requirements, duration of ventilation, ventricular dysfunction and early death^4 22^; consequently, it is the most common primary outcome measure in clinical trials of myocardial protection in children.^11^ While the rise in troponin is strongly correlated with the duration of ischaemia,^23^ it may be further elevated by surgical interventions, such as ventricular incision or resection.^24^

### Rationale

In this multicentre, phase II/III trial, we will test whether del Nido cardioplegia improves myocardial protection in children undergoing surgery for congenital heart disease compared with St. Thomas’ blood cardioplegia. In a recent UK and Ireland survey, we found that 29 (91%) respondent surgeons would be willing to use del Nido in a clinical trial, with the combination of del Nido and St. Thomas’ blood having the greatest acceptability.^15^ The trial was developed with advice from Professor Pedro del Nido and colleagues at Boston Children’s Hospital, who designed the eponymous solution and have the largest experience with its use in children in the world.^16^ Its pragmatic design was also informed by the survey findings, taking account of current UK practice on composition, temperature, dose and dosing interval, and noting concerns over the use of del Nido with an expected short cross-clamp time, thereby maximising its acceptability to the surgical community.^15^ del Nido cardioplegia will be made available for the first time in the UK through this trial, providing a unique and timely opportunity to address this important question. Once completed, it will be the largest clinical trial in paediatric cardiac surgery in the UK^25^ and the only multicentre trial of cardioplegia in children,^11^ with the potential to change routine clinical practice.

## Methods and analysis

### Design

The del Nido versus St. Thomas’ blood cardioplegia in the young (DESTINY) trial is a multicentre, patient-blinded and assessor-blinded, parallel-group, individually randomised controlled trial to compare the effects of two cardioplegia solutions on myocardial protection in children undergoing surgery for congenital heart disease at level 1 paediatric cardiac surgical centres in the UK (see Acknowledgements). It will be conducted through the Birmingham Clinical Trials Unit (BCTU), a UK Clinical Research Collaboration-registered clinical trials unit with expertise in surgical and paediatric trials. The full trial protocol is available as online supplemental appendix A.

### Inclusion and exclusion criteria

#### Inclusion criteria

All children (<16 years) undergoing cardiac surgery on CPB with cardioplegic arrest.

#### Exclusion criteria

The following children will be excluded from the study:

- Predicted cross-clamp time <30 min (eg, atrial septal defect, atrial septectomy, subaortic stenosis) at the discretion of the surgeon.
- Known contraindication to one of the constituents of either cardioplegia solution (eg, lidocaine/procaine hypersensitivity/allergy) or its method of delivery, including temperature (eg, haemoglobinopathy including sickle cell disease, cold agglutinins).
- Weight at the time of screening >50 kg.
- Ventricular assist device insertion/explant or transplantation.
- Preoperative inotropic support or extracorporeal life support (ECLS).
- Previous cardiac surgery with cardioplegic arrest within the last 30 days.
- Previous enrolment in the DESTINY trial.
- Emergency surgery, defined as required before the start of the next scheduled theatre list.
- Parent/guardian declines consent.

### Recruitment

**Figure 1:**
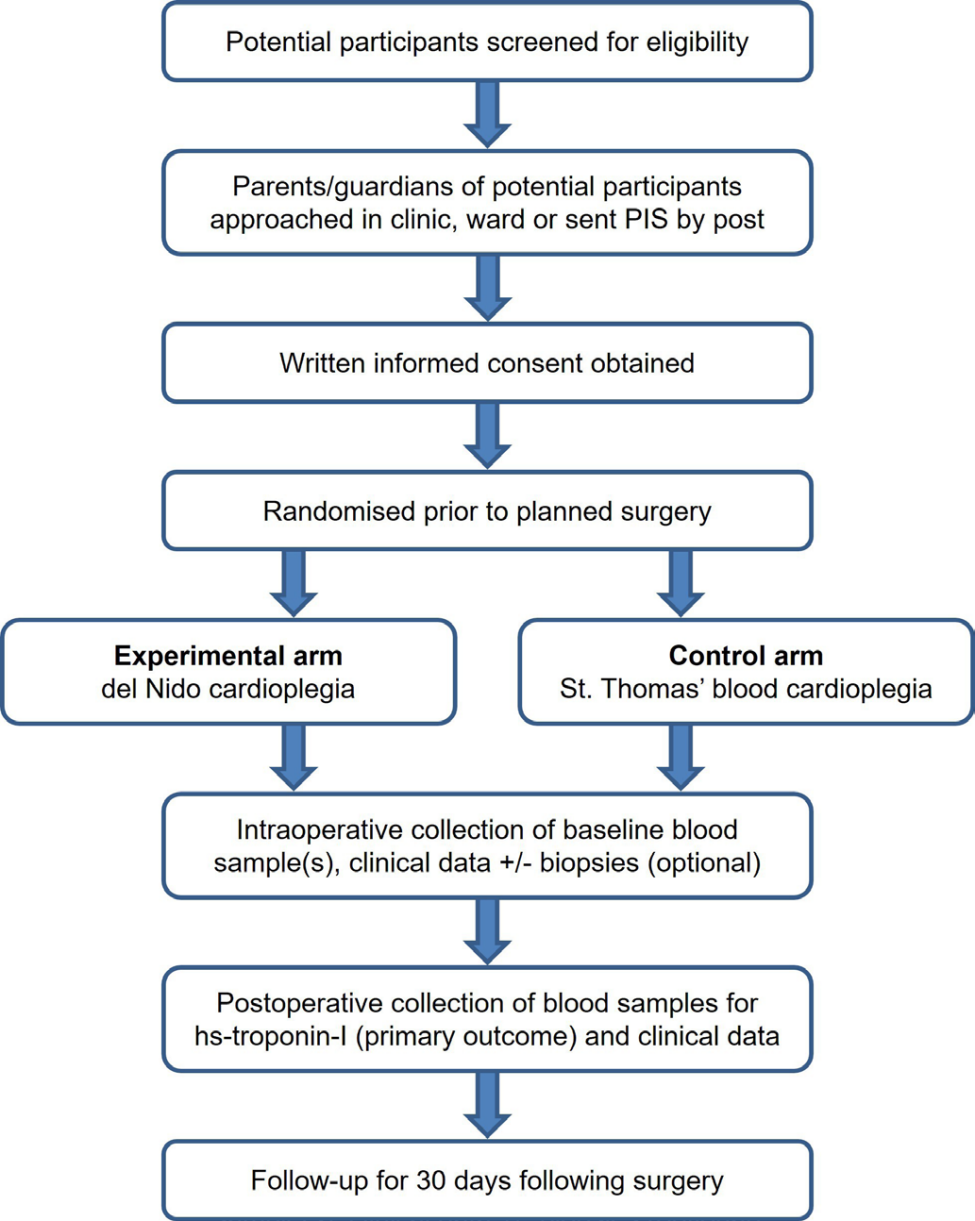
Participant pathway from screening to end of follow-up. PIS, parent/guardian information sheet.

All eligible patients will be identified from the multidisciplinary team meeting, surgical clinics or waiting lists by the patient’s direct clinical care team and their parents approached by a member of the clinical or research team. A parent/guardian information sheet (onlinesupplemental appendices B  C) will be provided, either in the clinic/ward or sent in the post, and they will be given at least 12 hours to consider their child’s participation and ask questions; children aged 8 years and above will also be given a child/young person information sheet, when appropriate (online supplemental appendix D). Written informed consent will be obtained by a suitably trained member of the research team prior to enrolment, with additional optional consent for substudies and future contact (onlinesupplemental appendices E  F). Children aged 8 years and above may also complete a child/young person assent form (online supplemental appendix G). The participant pathway through the trial is shown in figure 1.

### Randomisation

Prior to surgery, participants will be randomised in a 1:1 ratio to either del Nido or St. Thomas’ blood cardioplegia by a member of the local research team using a secure online randomisation system, with a minimisation algorithm incorporating the following factors:

- Age: neonate (0–30 days), infant (31 days<1 year), child (1<7 years), older child (7<16 years).
- Incision or resection of ventricular myocardium anticipated (yes/no).
- surgical centre.

To avoid any possibility of the allocation becoming predictable, a random element will be included in the algorithm. If online randomisation is unavailable, a telephone helpline with emergency paper randomisation will be used.

### Blinding

As the technique for delivery of cardioplegia and dosing interval differs between treatment groups, patient safety would be compromised by blinding those administering the cardioplegia. The operating surgeon, perfusionist, anaesthetist, theatre scrub team and research nurse therefore will not be blinded to the intervention. However, as the cardioplegia is only administered during surgery, patients, parents/guardians and outcome assessors such as cardiologists, other surgeons, intensive care unit (ICU) medical and nursing staff, and ward staff will be blinded to the allocation. This will be maintained by only documenting the use of cardioplegia in the medical notes without referring to which product was used.

Adherence to blinding will be rigorously maintained by concealing the perfusion chart, containing the treatment allocation and details of its delivery. At sites with a paper perfusion chart, this will be placed in a sealed envelope within the medical notes *before* leaving theatre. At sites with an electronic perfusion chart, access to the data will be restricted, and a printed copy placed in the sealed envelope. Allocation to the treatment will not be recorded in the patient’s notes. In the unlikely event of an emergency in which knowledge of the allocation may affect patient care or other safety reasons, the sealed envelope may be opened and the reason for opening recorded, and each site will be monitored. At patient handover to ICU following surgery, information on the type, dosing and timing of the cardioplegia will not be provided to the receiving team.

### Treatment arms

The trial interventions are either del Nido cardioplegia (experimental arm) or St. Thomas’ blood cardioplegia (control arm). Both investigational medicinal products (IMP) will be manufactured by Stockport Pharmaceuticals, a National Health Service (NHS) pharmaceutical manufacturer specialising in sterile IMP for clinical trials, and distributed to sites refrigerated by a specialist cold-chain courier. An Investigator’s Brochure for del Nido cardioplegia and Investigational Medicinal Product Dossier for both solutions have been approved by the regulator, the Medicines and Healthcare products Regulatory Agency (MHRA). The IMP will be supplied in 500 mL glass bottles, with the constituents shown in table 1, and the formulations and doses depicted in table 2, and each bottle visually inspected prior to release by a qualified person. The allocated cardioplegia solution will be administered by the clinical perfusionist, at the request of the operating surgeon, in an antegrade manner via an aortic root or direct coronary ostia cannula, following placement of a cross-clamp on the proximal aorta. The glass bottle containing the IMP will be vented during infusion, using a spike vent or similar. By placing a waste sucker into the right atrium after commencing its delivery, the systemic bioavailability of the cardioplegia constituents following passage through the coronary circulation will be markedly reduced. Adherence to treatment will be defined as receiving the allocated IMP for the index operation.

**Table 1:** Crystalloid composition of the experimental (del Nido) and control (St. Thomas’) treatments

del Nido	St. Thomas’ (Harefield preparation)
1 L Plasma-Lyte A base solution to which the following are added:	
Mannitol 20%, 16.3 mL	Sodium chloride 8.6 g
Magnesium sulphate 50%, 4 mL	Magnesium chloride 6.262 g
Sodium bicarbonate 8.4%, 13 mL	Calcium chloride 330 mg
Potassium chloride 2 mEq/mL, 13 mL	Potassium chloride 6.252 g
Lidocaine 1%, 13 mL	Procaine hydrochloride 1364 mg
Total volume: 1052.8 mL	In water for injection, per 1000 mL

**Table 2:** Formulation and dosing of cardioplegia in the experimental and control arms

Arm	IMP	Formulations	Dose
Experimental	del Nido cardioplegia	1:4 blood: crystalloid preparation	Initial dose of 20 mL/kg, subsequent doses every 60–90 min if required, at the discretion of the surgeon
Control	St. Thomas’ blood cardioplegia	4:1 blood: crystalloid using Harefield Hospital preparation	Initial dose of 20–30 mL/kg, subsequent doses of 15 mL/kg every 20–30 min, at the discretion of the surgeon

IMP, investigational medicinal products.

In patients who require repeat aortic cross-clamping during the index procedure (predicted to be <5%), the same cardioplegia solution should be used, if required, but the dose given will be at the discretion of the operating surgeon, that is, a smaller dose may be considered for an anticipated short period of repeat cross-clamping. In those who require an unplanned reoperation or planned procedure requiring cardioplegia within 30 days of the index procedure, the standard cardioplegia for the centre should be used rather than the IMP.

### Common aspects of care

#### Anaesthesia

Anaesthesia will be conducted at the discretion of the consultant anaesthetist and involve a balanced technique using volatile and intravenous anaesthesia and adjuncts, opioid pain relief and muscle relaxants. Routine monitoring will include continuous invasive arterial and central venous pressures, other cardiac output variables, urine output, blood gas analysis and near-patient clotting profile.^26^ Systemic anticoagulation will be achieved with heparin prior to the institution of CPB and reversed with protamine after the termination of CPB.

#### Surgery and perfusion

Repair of the congenital heart defect(s) will be performed following best clinical practice. After transfer to the operating room, the surgical checklist will be completed, the patient prepped and draped, and the chest opened through a median sternotomy. Standardised CPB will be established between the right atrium or venae cavae and the ascending aorta, with or without systemic hypothermia. An aortic cross-clamp will be applied to the proximal ascending aorta with antegrade cold cardioplegia given via the aortic root, with further doses given if required, as shown in table 2. Topical cooling of the heart with ice slush may be used at the discretion of the operating surgeon.

The first removal of the aortic cross-clamp with myocardial reperfusion will be considered as time zero for the recording of postoperative events. Following completion of the repair ±rewarming, CPB will be weaned and discontinued. In the event of difficulty separating from bypass or marked haemodynamic instability, subjective and objective measures of ventricular function will be obtained, and inotropic support instituted at the discretion of the operating team. Once haemodynamic stability and haemostasis have been achieved, the chest will be closed at the discretion of the surgical team and the patient transferred to the ICU. Standard postoperative care will proceed with anticipated removal of the arterial line at 12 hours following surgery, removal of the central line at 24 hours and transfer to the ward once routine ICU discharge criteria have been met. All postoperative decisions regarding escalation of therapy will be made by the blinded clinical team responsible for the care of the child without influence from the researchers.

### Trial investigations

The schedule for the intervention and collection of outcome data, blood and tissue samples is shown in online supplemental table S1.

#### Data collection

Clinical data will be collected by the research nurse before, during and after surgery. Inotrope usage in the first 48 hours will be used to generate a Vasoactive-Inotropic Score (VIS) (µg/kg/min).^27 28^ Arterial lactate and arterial/central venous oxygen saturations (for calculation of *omega*) will be recorded prior to surgery and at 3, 6, 9 and 12 hours. Duration of mechanical ventilation (hours) and length of stay in ICU (hours) and in hospital (days) following surgery will be documented.

#### Blood samples

Blood will be drawn from indwelling arterial or central venous lines at baseline (after induction of anaesthesia but prior to sternotomy) and at 3, 6, 9, 12 and 24 hours after reperfusion. Plasma samples for hs-troponin I (Abbott, Abbott Park, Illinois, USA) will be collected in paediatric EDTA tubes, centrifuged, split into two aliquots and stored at −80°C in remotely monitored freezers at each site until transfer for analysis at a core lab (Birmingham City Hospital, Birmingham or Midland Metropolitan University Hospital, Smethwick). Samples will be analysed in batches approximately every 6 months so that data on the primary outcome will be available to the data monitoring committee (DMC) prior to each meeting.

#### DNA samples

With specific consent, an additional 6 mL blood sample will be drawn into an EDTA tube at baseline. In the local laboratory, the buffy coat will be isolated, decanted and stored at −80°C until transfer in batches to the University of Birmingham, where DNA extraction will be performed, and it will remain stored at −80°C for genomic analysis.

#### Tissue samples

With specific consent, at sites with liquid nitrogen available, myocardial tissue will be obtained for a metabolism substudy. Right atrial samples will be taken soon after aortic cross-clamping (onset ischaemia) and just before its release (late ischaemia) to assess metabolic changes in the myocardium during the period of ischaemia. In patients undergoing routine resection of ventricular myocardium, such as hypertrophic septoparietal trabeculae from the right ventricular outflow tract or right ventricular free wall for anastomosis of a right ventricle to pulmonary artery conduit, these samples will be obtained during ischaemia when routinely resected. Specimens will each be briefly washed in ice-cold normal saline, placed in a cryotube, promptly snap-frozen in liquid nitrogen and stored at −80°C until transfer for metabolic phenotyping. Analysis of these samples is exploratory and will follow a separate analytical plan (see Substudy below).

### Outcome measures and follow-up

#### Primary outcome

Area under the time–concentration curve (AUC) for plasma high-sensitivity troponin I (μg/L/hour) in the first 24 hours after the index aortic cross-clamp release (reperfusion) as a marker of myocardial injury.

#### Secondary outcomes

- LCOS, defined as either of the following in the first 48 hours after reperfusion: VIS≥15,^27 28^ or major cardiac event (cardiac arrest, ECLS or death) (n).^29^
- Duration of mechanical ventilation (hours), defined as the number of hours from termination of index CPB to extubation.
- Length of postoperative stay on ICU (hours), defined as number of hours from admission to ICU from theatre following index procedure to discharge from ICU.
- Maximum VIS by thresholds: ≥10, ≥15 and ≥20 in the first 48 hours (n).
- Total VIS in the first 4 hours after ICU admission following the index procedure (score).
- Arterial lactate (mmol/L) in the first 12 hours.
- Omega, determined by (SaO_2_)/(SaO_2_-ScvO_2_) in the first 12 hours.^30^
- Total aortic cross-clamp time (min).
- Total volume of cardioplegia given (mL).
- Need for internal defibrillation during reperfusion (n).
- Delayed sternal closure, incidence (n) and duration (days).
- Unplanned reoperation, including chest reopening on ICU (n).
- Need for new renal replacement therapy (n), defined as the institution of either peritoneal dialysis or continuous venovenous haemofiltration, but not including a peritoneal dialysis catheter inserted intraoperatively and left on free drainage.
- Lowest estimated glomerular filtration rate, calculated using the bedside Schwartz equation and the peak postoperative creatinine on routine monitoring during the first 7 days following the index procedure (mL/min/1.73 m^2^), and assigned to a paediatric RIFLE (Risk, Injury, Failure, Loss, End-stage) stratum (n).^31^
- Length of postoperative stay in the hospital (days), defined as number of days from day of surgery to discharge from hospital or death, whichever is sooner.
- 30-day survival (n).

VIS will be calculated for each hour according to the highest dose of each drug received as an infusion during that hour^27 28^: dopamine (µg/kg/min)+dobutamine (µg/kg/min)+100×epinephrine (µg/kg/min)+10×milrinone (µg/kg/min)+10 000×vasopressin (units/kg/min)+100×norepinephrine (µg/kg/min).

#### Follow-up

Until 30 days after the index operation.

### Analysis

#### Sample size

It is hypothesised that del Nido cardioplegia reduces myocardial injury during surgery compared with St. Thomas’ blood cardioplegia. The DESTINY trial will use postoperative hs-troponin I release in the first 24 hours to measure the level of myocardial injury. The justification for the sample size is based on data from the Bilateral Remote Ischemic Conditioning in Children (BRICC) trial^32 33^; in a similar cohort of participants to those in the control group for this study, a mean AUC for postoperative hs-troponin release value of approximately 64.0 µg/L/hour and an SD of approximately 42.0 µg/L/hour were observed. To detect a difference of 30% (relative reduction, 19.2 µg/L/hour absolute reduction) between groups using the standard method of a two-sample t-test and assuming equal variance with 90% power and a type 1 error rate of 0.05, 102 participants per group will need to be randomised, 204 in total. Assuming and adjusting for a 3% loss to follow-up/dropout rate, 220 participants will need to be recruited.

#### Expected recruitment rate

The BRICC trial recruited young children undergoing surgery in Birmingham and Leeds with a recruitment rate of 86%^33^ and the Thermic-2 trial recruited 65% of eligible children in Bristol,^34^ which is similar to other published paediatric cardiac surgical trials (62–85%).^25 35 36^ As a clinical trial of an IMP, we anticipate that recruitment will be around 50–60%, and we will maintain a screening log to document exclusions and reasons given by parents who decline to participate.

#### Statistical analysis

The primary comparison groups will be composed of those treated with del Nido cardioplegia versus those treated with St. Thomas’ blood cardioplegia. In the first instance, all analyses will be based on the intention to treat principle. AUC for hs-troponin I release in the first 24 hours (µg/L/hour) will be calculated using the trapezoidal method, and adjusted mean differences along with 95% CIs will be estimated using a linear regression model. The variables used for the adjustment will be the minimisation parameters.

For secondary outcomes, continuous data items (eg, total aortic cross-clamp time) will be analysed using a linear regression model and results presented as adjusted mean difference and 95% CIs. Continuous outcomes measured across more than three time points (eg, arterial lactate, omega) will be analysed as repeated measures with linear mixed models using all available data. Baseline value of the measure (if available) and time by treatment interaction will be included with time as a continuous variable, in addition to all minimisation variables. Results will be presented as adjusted mean difference and 95% CIs.

Time to event data outcomes (eg, length of stay in ICU) will be analysed using the Cox regression model. Results will be presented as adjusted HR and 95% CIs. Kaplan-Meier plots will also be presented for visual interpretation.

Binary outcomes (eg, need for internal defibrillation during reperfusion) will be analysed using a logistic regression model. Adjusted risk ratios and risk differences along with 95% CIs will be derived using the marginal standardisation method.

Subgroup analyses on the primary outcome will be limited to the same variables used in the minimisation algorithm. Tests for statistical heterogeneity (eg, by including the treatment group by subgroup interaction parameter in the statistical model) will be performed prior to any examination of effect estimate within subgroups. The results of subgroup analyses will be treated with caution and will be used for the purposes of hypothesis generation only.

Every attempt will be made to collect full follow-up data on all study participants; it is thus anticipated that missing data will be minimal, especially given the short duration of follow-up. Randomised participants who do not undergo surgery cannot contribute data to the primary outcome and will therefore be excluded from the primary analysis. A detailed statistical analysis plan is under development and will be approved prior to database lock. The chief investigator and trial statisticians will have access to the final trial dataset.

### Adverse event reporting

Serious adverse event (SAE) reporting using standard definitions and practices for adult drug trials is not appropriate for clinical trials involving cardiac surgery in children,^37^ as events typically classified as SAEs occur commonly in the usual perioperative course for these children. Their use would lead to a significant reporting burden and may detract from those events which are considered critical. A recent UK multicentre study defined important early morbidities associated with paediatric cardiac surgery,^29^ which have been adopted by the UK National Congenital Heart Disease Audit.^38^ In the DESTINY trial, we therefore will use or adapt these definitions as the basis for expedited reporting of the following eight SAEs which are protocol defined as expected:

- Death.
- ECLS.
- Major adverse cardiac event.
- Unplanned reoperation/intervention in the early postoperative period.
- Acute neurological event.
- Sepsis, defined broadly as severe infection leading to life-threatening organ dysfunction.
- Deep surgical site infection.
- Other severe or life-threatening unexpected event.

If a patient fulfils more than one of the above SAE criteria, for example, cardiac arrest requiring ECLS, each event will be reported separately. If the patient has been discharged home within 30 days, the occurrence of any SAE within this timeframe will be ascertained either during a routine follow-up clinic visit or by a telephone call. Further details on the definitions, processes for SAE reporting, assessment of relatedness±expectedness, reporting to third parties and urgent safety measures are included in the full trial protocol (online supplemental appendix A).

Other events that are protocol defined as ‘expected’ will be considered as complications rather than adverse events, if they occur within 30 days of the index operation. These outcomes will be categorised by organ system, adapted from the Pediatric Heart Network’s Single Ventricle Reconstruction trial,^35 37^ and informed by the definitions developed during the Cardiac Morbidity study,^29^ as detailed in online supplemental appendix A.

### Management and oversight

#### Trial management group

The trialists will meet monthly to monitor all aspects of the trial, ensure that the protocol is adhered to, and take appropriate action to safeguard participants and the quality of the trial. During recruitment, the protocol may be amended considering emerging evidence, achievement of recruitment targets and feedback from trial sites.

#### Trial steering committee

An independent trial steering committee (TSC) will meet approximately every 6 months to provide oversight of the trial on behalf of the sponsor and funder, to ensure the safety of participants and integrity of the data. It will consider and act, as appropriate, on the recommendations of the DMC and ultimately carries the responsibility for deciding whether a trial needs to be stopped on grounds of safety or efficacy. Its duties and responsibilities are outlined in the TSC Charter, and membership of the committee is provided in the acknowledgements.

#### Data monitoring committee

An independent DMC will meet approximately every 6 months during recruitment to review unblinded efficacy and safety data, including on the primary outcome. Its duties and responsibilities are outlined in the DMC Charter, and membership of the committee is provided in the acknowledgements.

#### Data collection and management

All data will be entered onto the DESTINY trial database, a password-protected electronic database held on secure University of Birmingham servers for trial data with access limited to BCTU members of staff working on the trial. All paper case report forms will be stored securely in the research offices at recruiting NHS Trusts. Data will be semianonymised by removing non-essential potentially identifiable patient information; blood and tissue samples will be labelled with the unique trial ID number, date and time of collection. Adherence to trial processes will be audited by the independent clinical research compliance team at the University of Birmingham.

### Substudies

#### Qualitative

We will conduct semistructured interviews with surgeons and perfusionists to explore their experiences of using del Nido cardioplegia during the trial. The findings will inform the potential implementation of del Nido cardioplegia into routine NHS practice following the end of the trial.

#### Metabolic phenotyping

In a subset of patients, right atrial biopsies will be obtained soon after aortic cross-clamping (onset of ischaemia) and just before its release (late ischaemia) to assess metabolic changes in the myocardium during ischaemia. The impact of cardioplegia type on metabolism will be assessed through statistical analysis and pathway enrichment analysis.

#### Genetics

With additional funding, genomic analysis will be performed to assess potential associations with markers of myocardial protection during surgery.

#### Imaging follow-up

With additional funding, cardiac magnetic resonance (CMR) imaging and echocardiography will be used to assess late ventricular systolic and diastolic function at 5 years of age in a subgroup of patients aged 0–2 years at the time of surgery. Using core laboratory methodology to ensure data quality and standardisation, this will include non-invasive haemodynamic assessment and CMR tissue characterisation.

### Patient and public involvement (PPI)

PPI has been a central component in the development, conduct and planned reporting of this trial since its inception. The research question was informed by a focus group of parents in May 2017 which explored their perspectives on clinical trials in children and issues that would influence their willingness for their child participating in a trial of cardioplegia. One of these parents became a named PPI collaborator on the funding application, contributing to the proposal and plain English summaries.

The trial protocol was informed by our previous qualitative work on understanding parents’ decision-making on participation in clinical trials in children’s heart surgery.^39^ Parents of children who had previously undergone cardiac surgery at Bristol Royal Hospital for Children reviewed the draft parent information sheet and consent form, to improve clarity and readability for a lay audience. There are two parent representatives on the TSC, who are encouraged to actively participate in discussions and decision-making.

The outcomes of the trial will be communicated through a parent newsletter and national charity publicity, both of which will be coproduced with PPI collaborators. All PPI activities have been costed using the cost calculator according to National Institute for Health and Care Research (NIHR) Budgeting for Involvement,^40^ providing payment for time and expenses but not incentives.

## Ethics and dissemination

This clinical trial was approved by the West Midlands—Coventry and Warwickshire NHS Research Ethics Committee (21/WM/0149) on 30 June 2021, the MHRA (CTA: 21761/0367/001-0001, EudraCT: 2021-001915-10) on 4 July 2021 and the NHS Health Research Authority (279068) on 12 July 2021. It is sponsored by the University of Birmingham (RG_19–149) and registered on the NIHR Clinical Research Network portfolio (49735). The first patient was randomised on 11 February 2022 and recruitment is currently ongoing, with a planned completion date of 31 October 2025.

### Changes to the protocol since original ethical approval

Since the original ethical approval, four substantial amendments to the protocol have been sought and approved with the following significant changes:

- Amended randomisation procedure to increase flexibility in recruitment (August 2021).
- Added two exclusion criteria: ‘weight at time of surgery>50 kg’ and ‘previous enrolment in the DESTINY trial’. Added mechanism for concealing and restricting access to electronic perfusion charts. Amended del Nido IMP label, as per MHRA advice (August 2022).
- Removed the number of trial sites and names to enable expansion to additional sites. Amended St. Thomas’ IMP label, as per Good Manufacturing Practice instructions (October 2023).
- Amended exclusion criterion to ‘weight at time of screening>50 kg’ to better reflect process. Addition of qualitative substudy. Clarified how missing primary outcome data will be handled (February 2025).

### Dissemination plan

The findings of the clinical trial and substudies will be submitted for presentation at national and international meetings and manuscripts prepared for submission to leading journals. The authorship of the final trial report will include all members of the trial management group and named collaborators. The anonymised individual participant data collected during the trial will be available on request following publication of the study results.

Parents of children participating in the trial will be informed of the results through a newsletter. Children’s Heart Federation, the UK’s leading national children’s heart charity, has also agreed to publicise the outcomes of the trial on their website, newsletter, and social media, to reach a wide audience of those affected by congenital heart disease.

The first author is chief investigator of the trial and takes responsibility for the integrity of this protocol report, which adheres to the Standard Protocol Items: Recommendations for Interventional Trials recommendations.^41^ All authors have read and agree to the manuscript as written.

## Supplementary material

10.1136/bmjopen-2025-102029 online supplemental file 1

10.1136/bmjopen-2025-102029 online supplemental file 2

10.1136/bmjopen-2025-102029 online supplemental file 3

10.1136/bmjopen-2025-102029 online supplemental file 4

10.1136/bmjopen-2025-102029 online supplemental file 5

10.1136/bmjopen-2025-102029 online supplemental file 6

10.1136/bmjopen-2025-102029 online supplemental file 7

10.1136/bmjopen-2025-102029 online supplemental file 8

## References

[1] National Institute for Cardiovascular Outcomes Research National Congenital Heart Disease Audit (NCHDA). 2024 Summary Report (2020/21-2022/23).

[2] Buckberg GD, Brazier JR, Nelson RL (1977). Studies of the effects of hypothermia on regional myocardial blood flow and metabolism during cardiopulmonary bypass. J Thorac Cardiovasc Surg.

[3] Doenst T, Schlensak C, Beyersdorf F (2003). Cardioplegia in pediatric cardiac surgery: do we believe in magic?. Ann Thorac Surg.

[4] Mildh LH, Pettilä V, Sairanen HI (2006). Cardiac troponin T levels for risk stratification in pediatric open heart surgery. Ann Thorac Surg.

[5] Drury NE, Herd CP, Biglino G (2022). Research priorities in children and adults with congenital heart disease: a James Lind Alliance Priority Setting Partnership. Open Heart.

[6] Wittnich C, Peniston C, Ianuzzo D (1987). Relative vulnerability of neonatal and adult hearts to ischemic injury. Circulation.

[7] Bolling K, Kronon M, Allen BS (1996). Myocardial protection in normal and hypoxically stressed neonatal hearts: the superiority of hypocalcemic versus normocalcemic blood cardioplegia. J Thorac Cardiovasc Surg.

[8] Baker JE, Boerboom LE, Olinger GN (1988). Age-related changes in the ability of hypothermia and cardioplegia to protect ischemic rabbit myocardium. J Thorac Cardiovasc Surg.

[9] Imura H, Caputo M, Parry A (2001). Age-dependent and hypoxia-related differences in myocardial protection during pediatric open heart surgery. Circulation.

[10] del Nido PJ, Mickle DA, Wilson GJ (1988). Inadequate myocardial protection with cold cardioplegic arrest during repair of tetralogy of Fallot. J Thorac Cardiovasc Surg.

[11] Drury NE, Yim I, Patel AJ (2019). Cardioplegia in paediatric cardiac surgery: a systematic review of randomized controlled trials. Interact Cardiovasc Thorac Surg.

[12] Drury NE (2024). Myocardial protection in paediatric cardiac surgery: building an evidence-based strategy. Ann R Coll Surg Engl.

[13] Walcƶak A, Klein T, Voss J (2021). International Pediatric Perfusion Practice: 2016 Survey Results. J Extra Corpor Technol.

[14] Lao RX, Salvo C, Honjo O (2024). Cardioplegia practices in pediatric cardiovascular surgery. https://meeting.chss.org/Program/2024/A21.cgi.

[15] Drury NE, Horsburgh A, Bi R (2019). Cardioplegia practice in paediatric cardiac surgery: a UK & Ireland survey. Perfusion.

[16] Matte GS, del Nido PJ (2012). History and use of del Nido cardioplegia solution at Boston Children’s Hospital. J Extra Corpor Technol.

[17] Pourmoghadam KK, Ruzmetov M, O’Brien MC (2017). Comparing del Nido and Conventional Cardioplegia in Infants and Neonates in Congenital Heart Surgery. Ann Thorac Surg.

[18] Talwar S, Bhoje A, Sreenivas V (2017). Comparison of del Nido and St Thomas Cardioplegia Solutions in Pediatric Patients: A Prospective Randomized Clinical Trial. Semin Thorac Cardiovasc Surg.

[19] Doenst T, Borger MA, Weisel RD (2008). Relation between aortic cross-clamp time and mortality--not as straightforward as expected. Eur J Cardiothorac Surg.

[20] Ma M, Gauvreau K, Allan CK (2007). Causes of death after congenital heart surgery. Ann Thorac Surg.

[21] Gaies M, Pasquali SK, Donohue JE (2016). Seminal Postoperative Complications and Mode of Death After Pediatric Cardiac Surgical Procedures. Ann Thorac Surg.

[22] Immer FF, Stocker F, Seiler AM (1999). Troponin-I for prediction of early postoperative course after pediatric cardiac surgery. J Am Coll Cardiol.

[23] Taggart DP, Hadjinikolas L, Hooper J (1997). Effects of age and ischemic times on biochemical evidence of myocardial injury after pediatric cardiac operations. J Thorac Cardiovasc Surg.

[24] Su JA, Kumar SR, Mahmoud H (2019). Postoperative Serum Troponin Trends in Infants Undergoing Cardiac Surgery. Semin Thorac Cardiovasc Surg.

[25] Drury NE, Patel AJ, Oswald NK (2018). Randomized controlled trials in children’s heart surgery in the 21st century: a systematic review. Eur J Cardiothorac Surg.

[26] Checketts MR, Alladi R, Ferguson K (2016). Recommendations for standards of monitoring during anaesthesia and recovery 2015: Association of Anaesthetists of Great Britain and Ireland. Anaesthesia.

[27] Gaies MG, Gurney JG, Yen AH (2010). Vasoactive-inotropic score as a predictor of morbidity and mortality in infants after cardiopulmonary bypass. Pediatr Crit Care Med.

[28] Gaies MG, Jeffries HE, Niebler RA (2014). Vasoactive-inotropic score is associated with outcome after infant cardiac surgery: an analysis from the Pediatric Cardiac Critical Care Consortium and Virtual PICU System Registries. Pediatr Crit Care Med.

[29] Brown KL, Pagel C, Brimmell R (2017). Definition of important early morbidities related to paediatric cardiac surgery. Cardiol Young.

[30] Tibby SM, Murdoch IA (2003). Monitoring cardiac function in intensive care. Arch Dis Child.

[31] Akcan-Arikan A, Zappitelli M, Loftis LL (2007). Modified RIFLE criteria in critically ill children with acute kidney injury. Kidney Int.

[32] Drury NE, Bi R, Woolley RL (2020). Bilateral Remote Ischaemic Conditioning in Children (BRICC) trial: protocol for a two-centre, double-blind, randomised controlled trial in young children undergoing cardiac surgery. BMJ Open.

[33] Drury NE, van Doorn C, Woolley RL (2024). Bilateral remote ischemic conditioning in children: A two-center, double-blind, randomized controlled trial in young children undergoing cardiac surgery. JTCVS Open.

[34] Caputo M, Pike K, Baos S (2019). Normothermic versus hypothermic cardiopulmonary bypass in low-risk paediatric heart surgery: a randomised controlled trial. Heart.

[35] Ohye RG, Sleeper LA, Mahony L (2010). Comparison of shunt types in the Norwood procedure for single-ventricle lesions. N Engl J Med.

[36] McCrindle BW, Clarizia NA, Khaikin S (2014). Remote ischemic preconditioning in children undergoing cardiac surgery with cardiopulmonary bypass: a single-center double-blinded randomized trial. J Am Heart Assoc.

[37] Virzi L, Pemberton V, Ohye RG (2011). Reporting adverse events in a surgical trial for complex congenital heart disease: the Pediatric Heart Network experience. J Thorac Cardiovasc Surg.

[38] Healthcare Quality Improvement Partnership (2020). National Congenital Heart Disease Audit (NCHDA): 2020 summary report.

[39] Drury NE, Menzies JC, Taylor CJ (2021). Understanding parents’ decision-making on participation in clinical trials in children’s heart surgery: a qualitative study. BMJ Open.

[40] National Institute for Health and Care Research Learning for Involvement.

[41] Chan A-W, Tetzlaff JM, Altman DG (2013). SPIRIT 2013 statement: defining standard protocol items for clinical trials. Ann Intern Med.

