# Supplementary material for: del Nido versus St. Thomas’ blood cardioplegia in the young (DESTINY) trial: protocol for a multicentre randomised controlled trial in children undergoing cardiac surgery
Source: BMJ Open. 2025 Apr 14;15(4):e102029. doi: 10.1136/bmjopen-2025-102029 (PMC11997810; doi:10.1136/bmjopen-2025-102029)
Supplement: online supplemental file 1 [file bmjopen-15-4-s001.pdf]

TRIAL PROTOCOL

DESTINY Trial

del Nido versus St. Thomas' blood cardioplegia in the young (DESTINY) trial: a multi-centre randomised controlled trial in children undergoing cardiac surgery

This protocol has regard for the HRA guidance and is compliant with SPIRIT

**Version number:**	**5.0**
**Version date:**	14 February 2025

Protocol development

**Protocol Amendments**				
The following amendments and/or administrative changes have been made to this protocol since the implementation of the first approved version.				
**Amendment number**	**Date of amendment**	**Protocol version number**	**Type of amendment**	**Summary of amendment**
**1**	**19-Aug-2021**	**2.0**	**Substantial**	- This version of the protocol extends the time between randomisation and surgery to provide more flexibility with randomisation. In the *Trial Schema (section Trial Summary)* 'Randomised on day of planned surgery' changed to 'Randomised prior to planned surgery'.<br>- *Section 3.1.3 'Sub-studies' and Section 8.3 'Study procedures'*: Primary analysis of the biopsies will now be performed at University of Liverpool rather than Birmingham.<br>- *Section 7.1 'Intervention(s) and Schedule':* clarification is added on venting of the IMP glass bottles.<br>- *Section 7.6.2 'Accountability'*: DESTINY accountability logs will be provided to theatres as well as pharmacies. Only batch numbers will be used by the manufacturer, therefore 'Lot' is deleted from the text and provided likely reasons for disposal.<br>- *Section 8.3 'Study Procedures':* current version of the protocol states that with specific optional consent 2ml of blood will be collected from participant for genetic testing in the DESTINY genetic sub-study. Amendment is made to change the volume of the blood to 6ml.<br>- Correction of typographical errors.

**3**	**22-Aug-2022**	**3.0**	**Substantial**	- *Section 4.2 'Exclusion Criteria'*: Added two new exclusion criteria, 'Weight at the time of surgery >50kg' to limit use of multiple 500ml bottles of cardioplegia for one patient, and 'Previous enrolment in the DESTINY trial' to formally prevent recruited the same patient to the trial multiple times.<br>*Section 6.2.4 'Randomisation Process'*: Telephone service removed as no longer available.<br>- *Section 6.2.5 'Randomisation Records'*: Amended to new online DESTINY Participant Screening and Enrolment Log using REDCap.<br>- *Section 6.2.3 'Unblinding'*: Added mechanism for concealing and restricting access to electronic perfusion charts as sites no longer using paper charts.<br>- *Section 7.5.2 'Packaging and Labelling'*: 'Instruction for use' added to del Nido cardioplegia label, in line with MHRA advice.<br>- *Section 8.3 'Study procedures'*: Clarified the number and size of aliquots for troponin analysis and changed location of analysis to 'Birmingham City Hospital'.<br>- *Section 9.4 'Serious Adverse Event (SAE) Reporting in DESTINY'*: Added mechanism for determining SAE by telephone call.<br>- *Section 9.5 'SAEs not requiring expedited reporting to BCTU'*: Clarified that 'discharge' relates to hospital discharge.<br>- *Section 9.7.1 'Reporting procedure for expedited SAEs by sites'* and *Section 10.3.3 'Self-evident corrections'*: Amended in line with revised BCTU policies.<br>- Change of Trial Team Leader<br>- Addition of trial website address<br>- Correction of typographical errors.

**5**	**25-Oct-2023**	**4.0**	**Substantial**	- *Trial summary, Section 1.1.6 'Trial rationale' and Section 3.1.1 'Trial setting'*: Amended number of sites and removed names of sites to enable expansion to additional sites without specifically naming in the protocol.<br>- *Section 7.5.2 'Packaging and Labelling'*: 'Instruction for use' added to St Thomas' cardioplegia label as per requirements of EU GMP Annex 13: Investigational Medicinal Products instructions for use advice.<br>- *Section 8.3 'Study procedures; Blood sample for genetic analysis'*: amendment to instructions on processing buffy coat to reflect change in practice; and clarification that there is not currently funding for the analysis of extracted DNA but if funding is secured, these samples will be analysed. Changes requested following internal sponsor audit.<br>- *Section 14.5 'Data Monitoring Committee'*: Amended proposed frequency of committee meetings.<br>- Correction of typographical errors and administrative changes.

**10**	**14-Feb-2025**	**5.0**	**Substantial**	- *Funding and Support in Kind:* updated to reflect increase in funding.<br>-*Sponsor:* addition of named contact, change of department name and address.<br>-*Administrative Information:* updates to CI, DMC,TSC and TMG sections.<br>-*CI Signature Page:* expansion of *Compliance statement* to include 'the Human Tissue Act 2004'.<br>- *Trial Summary, Exclusion criteria:* 'Weight at time of surgery' amended to 'Weight at time of screening' to reflect process; also amended in *Section 4 Eligibility*<br>- *Trial Schema, Intraoperative collection* stage amended to reflect process: insertion of 'baseline blood sample(s), clinical data +/-'<br>- *Section 1.1.6 Trial Rationale*: deletion of outdated information re. prioritisation by centres and agreement to participation; and amendment to final justification/strength of the trial to reflect more direct potential benefit.<br>- *Section 3.1.3 Sub-studies*: addition of *Qualitative sub-study;* rationalisation of biopsy-related sub-studies due to number of samples collected.<br>- *Section 6.2.6 Informing Other Parties:* deletion of conditional 'If parents/guardians have agreed…' to reflect that consent to notification of participant's GP is mandatory for trial participation.<br>- *Section 8.3 Study procedures-* Blood samples for troponin analysis: more detail added to clarify reference timepoint for collection of samples in the case of more than one period of aortic cross-clamping; splitting of plasma samples for troponin analysis adjusted to 'two' to ensure sufficient volume for analysis;

				update to location of the laboratory performing troponin analysis.<br>- *Section 8.5 Participant Withdrawal and Changes of Status Within Trial:* clarification added re. data collection and inclusion in analyses for participants who are withdrawn prior to surgery.<br>- *Section 9.5 SAEs not requiring expedited reporting to BCTU, Cardiovascular*: in definition of *Impaired cardiac function*, time reference for Vasoactive Inotrope Score changed from '>48 hours' to 'at 48 hours' as sites are only collecting vasoactive inotrope data up to 48 hours.<br>- *Section 9.7.1 Reporting procedure for expedited SAEs by sites; 9.7.2 Provision of follow-up information:* and - *9.7.3 Assessment of relatedness:* amendments made to more accurately reflect the processes followed.<br>- *Section 10.4 Data Security:* update to scope of compliance to the *Data Protection Act (2018)* to include subsequent amendments.<br>- *Section 12 End of trial definition*: description of last data capture expanded to include resolution of data queries and completion of hs-troponin-I sample analysis; and timeframes for notifying MHRA, REC and RGT removed.<br>- *Section 13.2.1 Primary Outcome Measure:* correction made re. how missing baseline troponin values will be imputed by deletion of 'and type of defect undergoing repair'; and addition of description for handling of data if AUC for hs-troponin-I release in the first 24 hours (ng/L) is too skewed.<br>- *Section 13.2.4 Missing Data and Sensitivity Analyses:* amendment of wording re. randomising more participants to maintain sample size– 'will' changed to 'may'; exclusion of participants from primary analysis extended to those

				missing outcome data for one or more post aortic cross-clamp release timepoint, not solely at the 3- or 6-hour timepoints.<br>- *Section 22 Reference List*: Minor change.<br>- Correction of typographical and grammatical errors, deletion of erroneous repetitions of text, and minor changes to wording.

Funding and Support in Kind	
**Funder (s)**<br>(Names and contact details of all organisations providing funding and/or support in kind for this trial)	**Financial and non-financial support given:**
British Heart Foundation, Greater London House, 180 Hampstead Road, London NW1 7AW	£625,188
Funding Scheme (if applicable)	Clinical Study Grant
Funder's reference number	CS/20/3/34738
The funder had no role with respect to trial design; collection, management, analysis, and interpretation of data; writing of the report; and the decision to submit the report for publication.	

Protocol Sign Off

CI Signature Page

The undersigned confirm that the following protocol has been agreed and accepted and that the Chief Investigator agrees to conduct the trial in compliance with the approved protocol.

I agree to ensure that the information contained in this document will not be used for any other purpose other than the evaluation or conduct of the clinical investigation without the prior written consent of the Sponsor.

I also confirm that I will make the findings of the study publicly available through publication or other dissemination tools without any unnecessary delay and that an honest accurate and transparent account of the study will be given; and that any discrepancies from the study as planned in this protocol will be explained.

This protocol has been approved by:

Trial Name:	**DESTINY Trial**
Protocol Version Number:	Version: 5.0
Protocol Version Date:	14-Feb-2025
Chief Investigator:	Mr Nigel Drury
Trial Role:	Chief Investigator
Signature and date:	

Sponsor statement: By signing the IRAS form for this trial, the University of Birmingham, acting as sponsor confirm approval of this protocol.

Compliance statement: This protocol describes the DESTINY trial only. The protocol should not be used as a guide for the treatment of participants not taking part in the DESTINY trial.

The study will be conducted in compliance with the approved protocol, UK Policy Framework for Health and Social Care Research 2017, the Data Protection Act 2018, the Human Tissue Act 2004, and the principals of Good Clinical Practice as defined by the European Good Clinical Practice (GCP) Directive. Every care has been taken in the drafting of this protocol, but future amendments may be necessary, which will receive the required approvals prior to implementation.

**PI Signature Page**	
The undersigned confirm that the following protocol has been agreed and accepted and that the Principal Investigator agrees to conduct the trial in compliance with the approved protocol.<br>I agree to ensure that the confidential information contained in this document will not be used for any other purpose other than the evaluation or conduct of the clinical investigation without the prior written consent of the Sponsor.<br>This protocol has been approved by:	
Trial Name:	**DESTINY trial**
Protocol Version Number:	Version: 5.0
Protocol Version Date:	14-Feb-2025
PI Name:	
Name of Site:	
Signature and date:	________________________ __ __ / __ __ / __ __ __ __

**Reference Numbers**	
EudraCT number	2021-001915-10
Sponsor number	RG_19-149
ISRCTN reference number	ISRCTN13638147
IRAS reference number	279068

Sponsor	
University of Birmingham	Dr Birgit Whitman, Head of Research Ethics, Governance and Integrity
Contact Details:	Research Strategy & Services Division – Research Governance<br>Ash House, University of Birmingham, Edgbaston, Birmingham, B15 2SQ<br><br>Tel: 0121 414 3344<br>email: researchgovernance@bham.ac.uk

Administrative Information

**Chief Investigator**	
Mr Nigel Drury	Consultant in Paediatric Cardiac Surgery
Birmingham Women's and Children's NHS Foundation Trust, and Department of Cardiovascular Sciences, University of Birmingham, Birmingham	Tel: 0121 333 8731<br>email: nigel.drury@nhs.net

**Data Monitoring Committee – DMC**
Prof Steff Lewis, Chair of Medical Statistics, University of Edinburgh (chair)<br>Prof Mark G Hazekamp, Professor of Paediatric Cardiac Surgery, Leiden, the Netherlands<br>Dr Joseph C Manning, Professor of Nursing and Child Health, University of Leicester

**Trial Steering Committee – TSC**
Prof Jenny Kurinczuk, Professor of Perinatal Epidemiology, Oxford (chair)<br>Prof Carrol Gamble, Professor of Medical Statistics, Liverpool (statistician)<br>Dr David Inwald, Consultant Paediatric Intensivist, Addenbrooke's Hospital, Cambridge<br>Prof Enoch Akowuah, Professor and Consultant Cardiac Surgeon, JCUH, Middlesbrough<br>Mrs Sandra Ramsey (parent)<br>Mr David Land (parent)

**Trial Management Group – TMG**
Mr Nigel Drury – Chief Investigator<br>Mrs Manjinder Kaur – Trials Management Team Leader<br>Ms Ruth Evans – Trial Manager<br>Dr Kelly Handley – Senior Statistician<br>Dr Yongzhong Sun – Trial Statistician<br>Prof Massimo Caputo and Mr Timothy Jones – Surgical Representatives<br>Dr John Pappachan – PICU Representative<br>Paul Riley – Senior Analyst Programmer

**Trial Office Contact Details**	
The University of Birmingham, Edgbaston<br>Birmingham, B15 2TT	DESTINY@trials.bham.ac.uk
Randomisation website	https://www.trials.bham.ac.uk/DESTINY
Trial website	https://www.birmingham.ac.uk/DESTINY

ABBREVIATIONS

Abbreviation	Term
**ABPI**	Association of the British Pharmaceutical Industry
**ASD**	Atrial Septal Defect
**AUC**	Area Under the Curve
**BCTU**	Birmingham Clinical Trials Unit
**CI**	Chief Investigator
**CMR**	Cardiac Magnetic Resonance
**CPB**	Cardiopulmonary Bypass
**CRF**	Case Report Form
**CRN**	Clinical Research Network
**DCF**	Data Clarification Form
**DESTINY**	del Nido versus St. Thomas' blood cardioplegia in the young
**DMC**	Data Monitoring Committee
**ECLS**	Extra-Corporeal Life Support
**eGFR**	Estimated Glomerular Filtration Rate
**GCP**	Good Clinical Practice
**GP**	General Practitioner
**HES**	Hospital Episode Statistics
**HRA**	Health Research Authority
**ICF**	Informed Consent Form
**ISF**	Investigator Site File
**LCOS**	Low Cardiac Output Syndrome
**MHRA**	Medicines and Healthcare products Regulatory Agency
**mNCA**	Model Non-Commercial Agreement
**NHS**	National Health Service
**ONS**	Office for National Statistics
**PI**	Principal Investigator
**PICANet**	Paediatric Intensive Care Audit Network
**PICU**	Paediatric Intensive Care Unit

**PIS**	Participant Information Sheet
**QA**	Quality Assurance
**RCT**	Randomised Controlled Trial
**REC**	Research Ethics Committee
**RGT**	Research Governance Team
**SAE**	Serious Adverse Event
**SAR**	Serious Adverse Reaction
**SOP**	Standard Operating Procedure
**SUSAR**	Suspected Unexpected Serious Adverse Reaction
**TMF**	Trial Management File
**TMG**	Trial Management Group
**TSC**	Trial Steering Committee
**VAD**	Ventricular Assist Device
**VIS**	Vasoactive Inotrope Score

DEFINITIONS

Term	Abbreviation	Description
**Low cardiac output syndrome**	LCOS	Defined as *either* of the following in the first 48 hours after reperfusion: Vasoactive Inotrope Score (VIS) ≥15, or major cardiac event (cardiac arrest, ECLS or death).
**Renal replacement therapy**	RRT	Defined as the institution of either peritoneal dialysis or continuous venovenous haemofiltration, but not including a peritoneal dialysis catheter inserted intraoperatively and left on free drainage.
**Vasoactive inotrope score**	VIS	Calculated for each hour according to the highest dose of each drug received as an infusion during that hour.<br>VIS = dopamine (µg/kg/min)<br>+ dobutamine (µg/kg/min)<br>+ 100 x adrenaline (µg/kg/min)<br>+ 10 x milrinone (µg/kg/min)<br>+ 10,000 x vasopressin (units/kg/min)<br>+ 100 x noradrenaline (µg/kg/min)

TRIAL SUMMARY

Title: DESTINY: del Nido versus St. Thomas' blood cardioplegia in the young: a multi-centre randomised controlled trial in children undergoing cardiac surgery.

Objectives: To evaluate whether in children undergoing cardiac surgery with cardioplegic arrest, the use of del Nido cardioplegia, compared with St. Thomas' blood cardioplegia:

- reduces myocardial injury, as determined by AUC for plasma troponin following surgery

- reduces the duration of ischaemia (aortic cross-clamp time), the volume of cardioplegia given, and the need for internal defibrillation during reperfusion

- improves myocardial protection, reducing the frequency or severity of LCOS, and markers of reduced tissue perfusion (arterial lactate and *omega*)

- improves other clinical outcomes, including duration of mechanical ventilation, length of stay on the Paediatric Intensive Care Unit and in hospital, and 30-day survival

- impacts on changes in the metabolic profile of the myocardium during ischaemia

To determine whether in a UK multi-centre trial of cardioplegia in children, recruitment to time & target, collection of data & biological samples, and adherence to the trial protocol are feasible.

Trial Design: A phase II/III, 2 arm, multi-centre, patient- and assessor-blinded, parallel-group, individually randomised controlled trial.

Participant Population and Sample Size: 220 children undergoing cardiac surgery.

Setting: Level 1 paediatric cardiac surgery centres in the UK.

Eligibility Criteria:

Included patients will be:

- All children (<16 years) undergoing surgery with cardioplegic arrest

Exclusion criteria will be:

- Predicted cross-clamp time <30 minutes (e.g. atrial septal defect, atrial septectomy, sub-aortic stenosis) at the discretion of the Consultant surgeon
- Known contraindication to one of the constituents of either cardioplegia solution (e.g. lidocaine/procaine hypersensitivity/allergy) or its method of delivery, including temperature (e.g. haemoglobinopathy including sickle cell disease, cold agglutinins)
- Weight at the time of screening >50kg
- Ventricular assist device (VAD) insertion/explant or transplantation
- Preoperative inotropic support or extra-corporeal life support (ECLS)
- Previous cardiac surgery with cardioplegic arrest within the last 30 days
- Previous enrolment in the DESTINY trial
- Emergency surgery
- Parent/guardian declines consent

Interventions:

Experimental Arm: del Nido cardioplegia in a 1:4 blood:crystalloid preparation (section 7.1), given at 4-8°C, with an initial dose of 20ml/kg and subsequent doses every 60-90 minutes if required, at the discretion of the surgeon, as required.

Control Arm: St. Thomas' Hospital blood cardioplegia in a 4:1 blood:crystalloid using Harefield Hospital preparation (section 7.1), given at 4-8°C, with an initial dose of 20-30ml/kg, subsequent doses of 15 ml/kg every 20-30 minutes at the discretion of the surgeon, as required.

Outcome Measures:

Primary Outcome: Area under the time-concentration curve (AUC) for plasma high-sensitivity troponin-I (μg.h/L) in the first 24 hours after the index aortic cross-clamp release (reperfusion).

Secondary Outcomes:

- Low cardiac output syndrome (LCOS) defined as either of the following in the first 48 hours after reperfusion: Vasoactive Inotrope Score (VIS) ≥15 [1,2], or major cardiac event (cardiac arrest, ECLS or death) (n) [3]

- Duration of mechanical ventilation (hours), defined as the number of hours from termination of index CPB to extubation

- Length of postoperative stay on Paediatric Intensive Care (hours), defined as number of hours from admission to PICU from theatre following index procedure to discharge from PICU

- Max VIS by thresholds: ≥10, ≥15 and ≥20 in the first 48 hours (n)

- Total VIS in the first 4 hours after PICU admission following the index procedure (score)

- Arterial lactate (mmol/L) in the first 12 hours

- *Omega*, determined by [SaO_2]/[SaO_2-$ScvO_2$] in the first 12 hours [4]

- Total aortic cross-clamp time (mins)

- Total volume of cardioplegia given (ml)

- Need for internal defibrillation during reperfusion (n)

- Delayed sternal closure, incidence (n) and duration (days)

- Unplanned reoperation, including chest re-opening on PICU (n)

- Need for new renal replacement therapy (n)

- Lowest estimated glomerular filtration rate (eGFR), calculated using the bedside Schwartz equation and the peak postoperative creatinine on routine monitoring during the first 7 days following the index procedure (ml/min/1.73m^2), and according to the paediatric RIFLE categories (n) [5]

- Length of postoperative stay in the hospital (days), defined as number of days from day of surgery to discharge from hospital or death, whichever is sooner

- 30-day survival (n)

Trial Schema

The trial schema (***Figure 1***) shows a flowchart of the recruitment process the trial treatment and follow-up schedule.

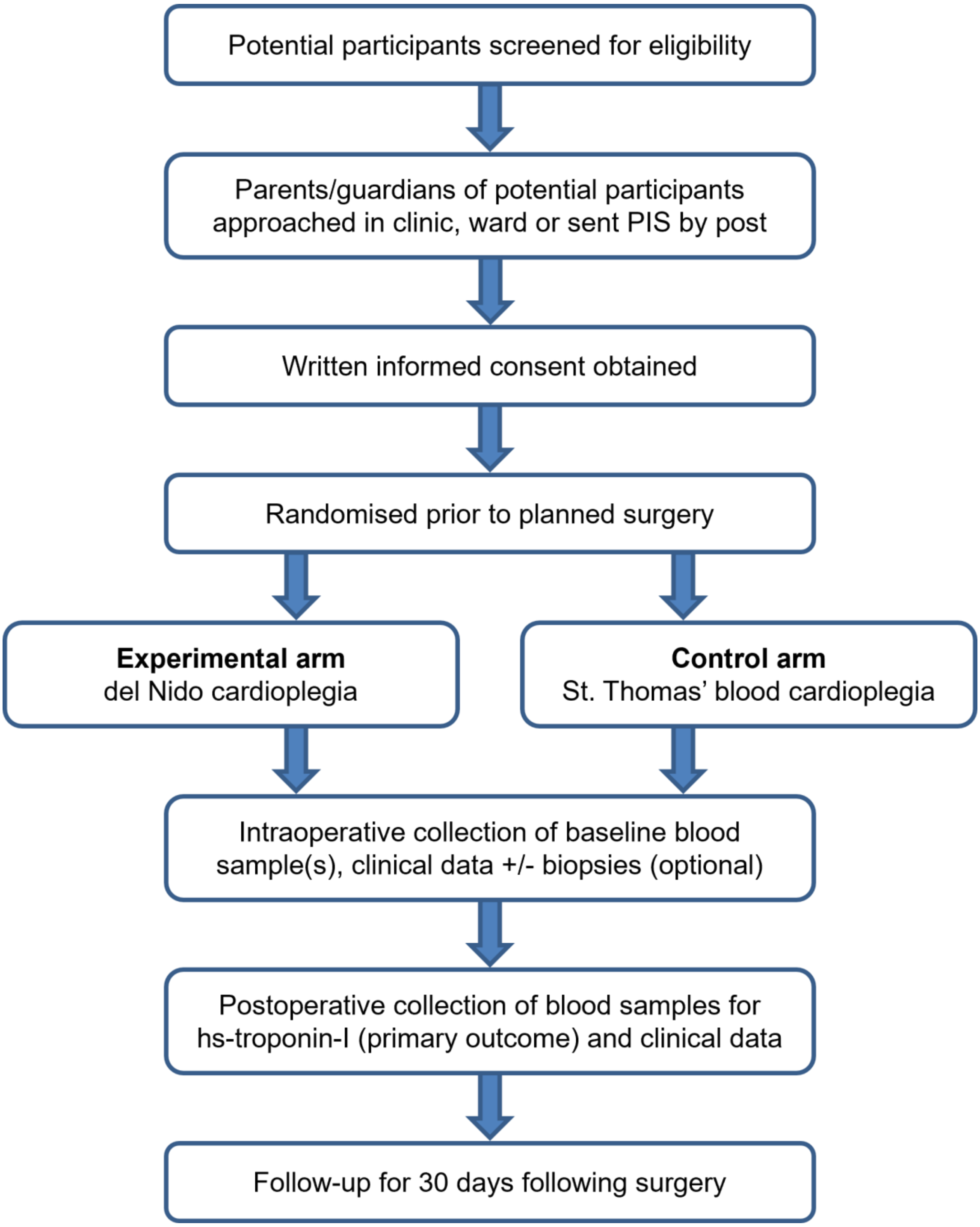

Figure 1. *Flowchart of the recruitment process from screening to follow up and the procedures and assessments to be performed*

TABLE OF CONTENTS

1 BACKGROUND AND RATIONALE 19
1.1 Background 19
1.1.1 Cardioplegia in paediatric cardiac surgery 19
1.1.2 Assessment of myocardial protection 19
1.1.3 Current evidence for del Nido cardioplegia in children 20
1.1.4 Variations in paediatric cardioplegia practice in UK & Ireland 20
1.1.5 Acceptability of a cardioplegia trial to parents 21
1.1.6 Trial Rationale 21
1.1.7 Justification for participant population 21
1.1.8 Justification for design 21
1.1.9 Choice of intervention 22
2 AIMS AND OBJECTIVES 23
2.1 Main Trial Objectives 23
2.1.1 Clinical Aims and Objectives 23
2.1.2 Mechanistic Aims and Objectives 23
3 TRIAL DESIGN AND SETTING 24
3.1 Trial Design 24
3.1.1 Trial Setting 24
3.1.2 Identification of participants 24
3.1.3 Sub-studies 24
3.1.4 Assessment of Risk 25
4 ELIGIBILITY 26
4.1 Inclusion Criteria 26
4.2 Exclusion Criteria 26
4.3 Co-enrolment 26
5 CONSENT 27
6 ENROLMENT AND RANDOMISATION 29
6.1 Enrolment and Screening 29
6.2 Randomisation 29
6.2.1 Randomisation Methodology 29
6.2.2 Blinding 29
6.2.3 Unblinding 30
6.2.4 Randomisation Process 30
6.2.5 Randomisation Records 30
6.2.6 Informing Other Parties 30
7 TRIAL TREATMENT / INTERVENTION 31
7.1 Intervention(s) and Schedule 31
7.2 Drug Interaction or Contraindications 32
7.3 Treatment Modification 32

7.4 Cessation of Treatment / Continuation after the Trial 32
7.5 Treatment Supply and Storage 32
7.5.1 Treatment Supplies 32
7.5.2 Packaging and Labelling 32
7.5.3 Drug Storage 33
7.6 Accountability and Compliance Procedures 33
7.6.1 Compliance 33
7.6.2 Accountability 33
8 OUTCOME MEASURES AND STUDY PROCEDURES 34
8.1 Primary Outcome Measure 34
8.2 Secondary Outcome Measures 34
8.3 Study procedures 35
8.4 Schedule of Assessments 36
8.5 Participant Withdrawal and Changes of Status Within Trial 37
9 ADVERSE EVENT REPORTING 38
9.1 Definitions 38
9.2 Adverse Events - General Recording Requirements 39
9.3 Adverse Event Reporting in DESTINY 39
9.4 Serious Adverse Event (SAE) Reporting in DESTINY 39
9.5 SAEs not requiring expedited reporting to BCTU 39
9.6 Events that require expedited reporting to the Sponsor on the SAE Form 41
9.7 Reporting procedure 41
9.7.1 Reporting procedure for expedited SAEs by sites 41
9.7.2 Provision of follow-up information 42
9.7.3 Assessment of relatedness 42
9.8 Assessment of Expectedness by the CI 43
9.9 Protocol defined expected SAEs 44
9.10 Reporting SAEs to third parties 44
9.10.1 Reporting to the Competent Authority and main Research Ethics Committee 44
9.10.2 Reporting to the Data Monitoring Committee 44
9.11 Urgent Safety Measures 44
9.12 Monitoring Pregnancies for Potential Serious Adverse Events 44
10 DATA HANDLING AND RECORD KEEPING 45
10.1 Source Data 45
10.2 Case Report Form (CRF) Completion 45
10.3 Data Management 47
10.3.1 Source Data 47
10.3.2 Data Clarification 47
10.3.3 Self-evident corrections 47
10.4 Data Security 48
10.5 Archiving 48

11 QUALITY CONTROL AND QUALITY ASSURANCE 49
11.1 Site Set-up and Initiation 49
11.2 Monitoring 49
11.3 Onsite Monitoring 49
11.4 Central Monitoring 49
11.5 Audit and Inspection 49
11.6 Notification of Serious Breaches 50
12 END OF TRIAL DEFINITION 50
13 STATISTICAL CONSIDERATIONS 51
13.1 Sample Size 51
13.2 Analysis of Outcome Measures 51
13.2.1 Primary Outcome Measure 51
13.2.2 Secondary Outcome Measures 52
13.2.3 Subgroup Analyses 52
13.2.4 Missing Data and Sensitivity Analyses 52
13.3 Planned Interim Analysis 52
13.4 Planned Final Analyses 52
14 TRIAL ORGANISATIONAL STRUCTURE 53
14.1 Sponsor 53
14.2 Coordinating Centre 53
14.3 Trial Management Group 53
14.4 Trial Steering Committee 53
14.5 Data Monitoring Committee 53
14.6 Finance 54
15 ETHICAL CONSIDERATIONS 54
16 CONFIDENTIALITY AND DATA PROTECTION 55
17 FINANCIAL AND OTHER COMPETING INTERESTS 55
18 INSURANCE AND INDEMNITY 55
19 POST-TRIAL CARE 56
20 PUBLICATION POLICY 56
21 ACCESS TO FINAL DATA SET 56
22 REFERENCE LIST 57

1 BACKGROUND AND RATIONALE

1.1 Background

1.1.1 Cardioplegia in paediatric cardiac surgery

During most surgery for congenital heart disease, it is necessary to stop the heart, allowing access to a still and bloodless field to enable repair of intracardiac defects. Cardioplegia has been fundamental to arresting the heart and protecting against ischaemia-reperfusion (IR) injury during surgery for over 40 years, with approx. 3,500 cardiac surgical operations with cardioplegic arrest performed in children in the UK & Ireland each year [6]. Whilst on cardiopulmonary bypass, a cross-clamp is placed across the proximal aorta and cardioplegia injected into the coronary arteries via the aortic root, leading to electro-mechanical arrest. This reduces myocardial oxygen uptake to only 10% of that of the perfused beating heart, and progressive hypothermia leads to a further stepwise reduction [7]. However, myocardial injury still occurs *routinely* following aortic cross-clamping in children, as demonstrated by the ubiquitous release of troponin after surgery [8,9]. Myocardial protection therefore is a key determinant of heart function and outcome following cardiac surgery.

Current paediatric cardioplegia techniques are primarily derived from adult or laboratory models; however, the immature myocardium has significant structural, physiological, and metabolic differences from the adult heart, including sarcoplasmic reticulum development, mitochondrial density, substrate utilisation, calcium handling and antioxidant defences [3]. It is less tolerant of ischaemia and more sensitive to Ca^{2+} overload-mediated injury during reperfusion, particularly with hypoxaemia [10,11]. Myocardial protection that is effective in adults therefore may not be optimal for young children [12,13], especially neonates and those with chronic preoperative cyanosis [14].

Many types of cardioplegia solution are available and there is wide variation in their use worldwide [15]. In the US, del Nido cardioplegia is the most used in children [16] with a recent survey finding it is preferred by 76% of centres performing complex surgery in neonates [17]. On the other hand, St. Thomas' blood cardioplegia is used by most surgeons in most centres in the UK [18] where del Nido solution is not currently commercially available. There are significant theoretical and practical differences between these two autologous blood cardioplegia solutions; identifying the best cardioplegia for specific patient groups will enable the care of the child undergoing surgery to be individualised, potentially improve outcomes by reducing myocardial injury, morbidity, and costs, and may improve long-term cardiac function.

1.1.2 Assessment of myocardial protection

Low cardiac output syndrome (LCOS) in the early postoperative period reflects the degree of myocardial injury and the need for inotropic support to maintain adequate tissue perfusion. The presence of LCOS is a major determinant of outcome after heart surgery in children and most deaths in the early postoperative period are attributed to a low cardiac output [19,20]. However, there are no widely accepted methods for directly measuring cardiac output in young children and therefore the determination of low cardiac output is dependent upon other clinical measures such as need for inotropic support.

Postoperative elevation of plasma troponin is a marker of myocardial injury and has been shown to strongly correlate with clinical outcomes including inotropic support, duration of ventilation, ventricular dysfunction and early death [9,21] consequently, it is the most common

primary outcome measure in clinical trials of myocardial protection in children [22]. Whilst the rise in troponin is strongly correlated with duration of ischaemia [23], it may be further elevated by the surgical intervention, such as ventricular incision or muscle resection [24].

1.1.3 Current evidence for del Nido cardioplegia in children

In a recent systematic review of randomised controlled trials of cardioplegia in children, we identified 26 studies that were exclusively single centre, phase II trials, recruiting few patients (median 48, IQR 30-99) and at risk of systematic bias [22]. The most frequent comparison was blood versus crystalloid in 10 trials, with only two comparing del Nido with St. Thomas' blood cardioplegia. The most common endpoints were biomarkers of myocardial injury (17, 65%), inotrope requirements (15, 58%) and length of stay in PICU (11, 42%). However, the heterogeneity of patients, interventions and reported outcome measures prohibited meta-analysis. Of concern, these trials included only 21 neonates, a high-risk group in whom the effects of cardioplegia are less well understood [8]. We concluded that the current literature contains no late phase trials and the small size, inconsistent use of endpoints and low quality of reported trials provides a limited evidence-base for patient care; the best cardioplegia solution for children remains unknown.

Several recent phase II trials have compared del Nido v St. Thomas' blood (or similar) cardioplegia in children, all small studies in middle income countries. Talwar et al. randomised 100 patients ≤12 years undergoing tetralogy of Fallot or Ventricular Septal Defect repair in New Delhi; they found a lower troponin release, higher cardiac index, shorter cross-clamp time, duration of ventilation, ICU and hospital stay in the del Nido group [25]. Elsewhere in India, both Panigrahi et al. and Negi et al. found lower inotrope requirements and lower cardioplegia dose with del Nido in 60 and 56 children, respectively but no difference in troponin or creatine kinase release [26,27]. Rushel et al. found lower troponin immediately and at 24 hours after repair of tetralogy of Fallot in 60 children in Bangladesh, associated with reduced cross-clamp time, duration of ventilation and ICU stay [28]. On the other hand, Gorjipour et al. found no difference in troponin in 59 children undergoing surgery in Iran [29]. These trials all have significant methodological issues, including poor design, lack of sample size calculation, inadequate or no blinding, with a high or unclear risk of bias [22], and recruited no neonates but represent the only RCT-level evidence available for del Nido cardioplegia in children.

Two retrospective observational studies compared the use of del Nido with other blood cardioplegia in children. Buel et al. found a 6-fold reduction in the need for internal defibrillation during reperfusion after switching from St. Thomas' to del Nido (26.8% v 4.4%, $p<0.001$), with the greatest reduction in those weighing <6kg (17.5% v 1.2%, $p<0.001$), who were mostly neonates [30]. O'Brien et al. found that del Nido was associated with lower troponin release (0.83 v 13.8μg/L, $p<0.001$) compared with a matched cohort who received modified Buckberg blood cardioplegia [31].

1.1.4 Variations in paediatric cardioplegia practice in UK & Ireland

In a recent survey of practice, we received responses from 32 (78%) Consultant paediatric cardiac surgeons and all 12 Chief perfusionists in the UK & Ireland [18]. We found that St. Thomas' blood (Harefield preparation) is used routinely by 19 (59%) surgeons from 8 (67%) centres, with another 7 (22%) using a similar blood cardioplegia; no centre uses del Nido. 29 (91%) surgeons would be willing to use del Nido in a trial, with the combination of del Nido and St. Thomas' blood having greatest acceptability, but 5 (17%) expressed concern over its use with an expected short cross-clamp time. Only 2 (6%) were not willing to change their

practice within a trial setting, both of whom have since retired. Chief perfusionists reported that no additional equipment other than disposables would be required. Responses on the composition, temperature, dose, and dosing interval have also informed the pragmatic trial design, thereby maximising its acceptability to the surgical community.

1.1.5 Acceptability of a cardioplegia trial to parents

We invited parents of children who have previously undergone cardiac surgery to take part in a focus group to explore their perspectives on clinical trials and thoughts on participation in a cardioplegia trial. They would be keen for their child to participate in a trial if: it may benefit their child or others in the future; the drug is already proven and in use elsewhere; it is not going to cause harm; and their surgeon thinks the study is a good idea. They were particularly in favour of comparing the ‘standard treatments’ used in UK and US.

As outlined above, there are both a lack of evidence from late phase trials to support clinical decision-making [17] and variations in practice across the UK & Ireland [18], suggesting the presence of clinical equipoise; this is confirmed by the willingness of almost all surgeons to change their practice within a trial. Acceptability to both parents and healthcare professionals supports the feasibility of this multi-centre clinical trial.

1.1.6 Trial Rationale

This multi-centre phase II/III trial aims to determine whether del Nido cardioplegia improves myocardial protection in children compared with St. Thomas’ blood cardioplegia. It was developed with advice from Prof Pedro del Nido & colleagues at Boston Children’s Hospital and Harvard University, who designed the eponymous solution and have the largest experience with its use in children in the world. The pragmatic design has been informed by our recent survey of practice and willingness to randomise within a trial [18], thereby maximising the potential impact of the trial on the surgical community. del Nido cardioplegia will be made available for the first time in the UK through this trial, providing a unique and timely opportunity to address this important question. Once completed, it will be the largest clinical trial in paediatric cardiac surgery in the UK [32] and the only multi-centre trial of cardioplegia in children [22], with the potential to change routine clinical practice in the UK and beyond.

1.1.7 Justification for participant population

The inclusion criteria are deliberately wide to ensure the generalisability of results. In the absence of more precise data concerning the effects of cardioplegia on the young heart, the commonly accepted societal definition of a child, <16 years of age, has been applied.

1.1.8 Justification for design

Randomised controlled trials are considered the ‘gold standard’ for evidence-based medicine. Because of the nature of the procedure it is not possible to conceal the team performing the procedure from the allocation (see section 6.2.2 for details) but they will not know in advance which of the allocated treatments will be received and therefore selection bias will be removed.

1.1.9 Choice of intervention

del Nido cardioplegia is unique in that it was developed specifically for protection of the paediatric myocardium [33]. Like St. Thomas', it is a modified depolarising solution that causes arrest by elevating extracellular potassium but it provides additional cellular protection through: lidocaine, a Na^+ channel blocker that prevents intra-cellular Na^+ and Ca^{2+} accumulation during arrest [26] and increases the refractory period of the cardiac myocyte; mannitol, an oxygen free radical scavenger that has osmotic effects to reduce myocardial oedema [34]; and a lower proportion of autologous whole blood, which maintains physiological buffering via erythrocyte carbonic anhydrase [33] but has only trace Ca^{2+} concentration, reducing myocardial Ca^{2+} accumulation during ischaemia [35].

There is extensive laboratory data to support the principle of del Nido cardioplegia for the immature myocardium. In a neonatal piglet model, Bolling et al demonstrated the superiority of a hypocalcaemic blood cardioplegia in hypoxic hearts, with better preservation of ventricular function and energetics [6]. In rat hearts, van Emous showed that lidocaine reduced Na^+ influx during ischaemia leading to improved functional and metabolic recovery [36]. In large animal models, del Nido's group demonstrated the specific benefits of this highly buffered, low-calcium, glycolysis-promoting solution in neonates over standard hyperkalaemic solutions, with improved myocardial contractility and oxidative metabolism [37,38]. del Nido cardioplegia is thereby customised to reduce the impact of ischaemia-reperfusion on the immature myocardium, leading to its increasing popularity in the US [16,17] but with little clinical trial evidence of translation into improved clinical outcomes.

Our group has studied the impact of cardioplegia type on myocardial metabolism in a Langendorff mouse model. Using ultra-performance liquid chromatography-mass spectrometry (UPLC-MS), we showed clear metabolic differences during ischaemia between hearts protected with del Nido versus St. Thomas' blood cardioplegia suggesting a metabolic basis for any difference in myocardial protection afforded by the two solutions [39].

del Nido cardioplegia also has a specific and important practical advantage in that the usual interval between doses is much longer (60-90 mins) than for St. Thomas' (20-30 mins), such that in most cases, only a single dose is required. Eliminating or reducing the need to interrupt surgical flow to re-dose with cardioplegia improves the efficiency of the operation [40] and has been shown to shorten the overall burden of ischaemia (cross-clamp time) [25]. Duration of aortic cross-clamp remains an independent predictor of outcome after surgery [40] and therefore purely by reducing the duration of ischaemia, del Nido cardioplegia might be expected to reduce the impact on the myocardium and potentially improve outcomes.

2 AIMS AND OBJECTIVES

2.1 Main Trial Objectives

2.1.1 Clinical Aims and Objectives

To evaluate whether in children undergoing cardiac surgery with cardioplegic arrest, the use of del Nido cardioplegia, compared with St. Thomas' blood cardioplegia:

- reduces myocardial injury, as determined by AUC for plasma troponin following surgery

- reduces the duration of ischaemia (aortic cross-clamp time), the volume of cardioplegia given, and the need for internal defibrillation during reperfusion

- improves myocardial protection, reducing the frequency or severity of LCOS, and markers of reduced tissue perfusion (arterial lactate and *omega*)

- improves other clinical outcomes, including duration of mechanical ventilation, length of stay on the Paediatric Intensive Care Unit and in hospital, and 30-day survival

2.1.2 Mechanistic Aims and Objectives

To determine whether in children undergoing cardiac surgery with cardioplegic arrest, the use of del Nido cardioplegia, compared with St. Thomas' blood cardioplegia, impacts on changes in the metabolic profile of the myocardium during ischaemia.

3 TRIAL DESIGN AND SETTING

3.1 Trial Design

The DESTINY trial is a phase II/III, 2 arm, multi-centre, participant-blinded, assessor-blinded, parallel-group, individually randomised controlled trial with allocations on a 1:1 basis.

3.1.1 Trial Setting

The trial will take place at a number of Level 1 paediatric cardiac surgery centres in the UK, with the potential to expand to more centres in the event of local issues delaying set-up or restricting recruitment once open.

3.1.2 Identification of participants

At the participating centres potential participants fulfilling the inclusion/exclusion criteria will be identified from the multi-disciplinary team meeting, surgical waiting list, clinic, or ward by the patient's direct clinical care team, i.e. Consultant or Specialist Nurse. Electronic and/or paper healthcare records will be used to identify suitable patients and determine eligibility.

Following identification by the clinical team, the potential participant will then be referred to the DESTINY research team. The parent/guardian(s) +/- child will be approached by a delegated member of the clinical or research team. The Patient Information Sheet (PIS) will be provided either in person or by post and the parent/guardian(s) will be given sufficient time to read the PIS, consider participation and ask questions (see section 5).

In those willing to participate in the trial, written informed consent will be obtained by a member of the research team who will have up to date GCP training, in depth knowledge of the study protocol and have been delegated authority from the local Principal Investigator. Their name will be recorded on the **DESTINY Site Signature and Delegation Log**. Upon obtaining of informed consent, baseline data will be collected.

The outcome of the conversation, i.e. whether the parent/guardian(s) agreed to participation, have given informed consent, and have been randomised to the trial will be recorded in the trial screening log. This will be necessary for assessing recruitment feasibility and for future conversations with the patient should they wish to have more time to consider participation to the trial.

3.1.3 Sub-studies

The trial intends to incorporate several potential sub-studies:

- **Qualitative sub-study**: Semi-structured interviews with surgeons and perfusionists to explore their experiences of using del Nido cardioplegia during the trial, to inform its potential implementation into routine NHS practice at the end of the trial.

- **Metabolic sub-study**: In a subset of patients, right atrial biopsies will be obtained soon after aortic cross-clamping (onset ischaemia) and just before its release (late ischaemia) to assess metabolic changes in the myocardium during ischaemia. The impact of cardioplegia type on metabolism will be assessed through pathway enrichment analysis, in collaboration with University of Liverpool.

- **Genetics sub-study**: With additional funding, genomic analysis will be performed to assess potential associations with markers of myocardial protection during surgery.

- **Imaging follow-up sub-study**: With additional funding, cardiac magnetic resonance (CMR) imaging and echocardiography will be used to assess late ventricular systolic and diastolic function at 5 years of age in a sub-group of patients aged 0-2 years at the time of surgery. Using core laboratory methodology to ensure data quality and standardisation, this sub-study will include non-invasive haemodynamic assessment and CMR tissue characterisation.

3.1.4 Assessment of Risk

All clinical trials can be considered to involve an element of risk and, in accordance with BCTU operating procedures, this trial has been risk assessed, to clarify any risks relating uniquely to this trial. This risk assessment, on the basis that the IMP is not licensed within the EU, but there is extensive clinical evidence on its use in this patient group in both the USA and Spain, concluded that this trial is:

- Type B = Somewhat higher than that of standard medical care.

4 ELIGIBILITY

4.1 Inclusion Criteria

Children (<16 years) undergoing cardiac surgery on cardiopulmonary bypass with cardioplegic arrest.

4.2 Exclusion Criteria

- Predicted cross-clamp time <30 minutes (e.g. atrial septal defect, atrial septectomy, sub-aortic stenosis) at the discretion of the Consultant surgeon
- Known contraindication to one of the constituents of either cardioplegia solution (e.g. lidocaine/procaine hypersensitivity/allergy) or its method of delivery, including temperature (e.g. haemoglobinopathy including sickle cell disease, cold agglutinins)
- Weight at the time of screening >50kg
- Ventricular assist device (VAD) insertion/explant or transplantation
- Preoperative inotropic support or extra-corporeal life support (ECLS)
- Previous cardiac surgery with cardioplegic arrest within the last 30 days
- Previous enrolment in the DESTINY trial
- Emergency surgery
- Parent/guardian declines consent

4.3 Co-enrolment

Co-enrolment into cluster randomised trials is permitted, but not into any individually randomised controlled trial unless specifically agreed with the Trial Management group (TMG) in advance.

5 CONSENT

It will be the responsibility of the Principal Investigator or their delegate to obtain written informed consent for each participant prior to performing any trial related procedure. Research Nurses may be permitted to obtain informed consent if local practice allows, and this responsibility has been delegated by the Principal Investigator (PI) as captured on the Site Signature and Delegation Log.

A Parent/Guardian Participant Information Sheet (PIS) will be provided to facilitate this process. There are two versions of the PIS: *version a* is for use at sites with liquid nitrogen available to snap-freeze tissue biopsies and includes information on taking biopsies; *version b* is for use at sites without liquid nitrogen availability and does not include information on taking biopsies as this will not be performed at such sites. In addition, a Child/Young Person PIS will be provided to children aged 8 years and above, when appropriate. The Principal Investigator or their delegate will ensure that they adequately explain the aim, trial intervention, anticipated benefits, and potential hazards of taking part in the trial to the parent/guardian +/- participant. They will also stress that participation is voluntary and that they are free to decline to take part or may withdraw from any aspect of the trial at any time.

The parent/guardian +/- participant will be given adequate time (at least 12 hours) to read the PIS and to discuss their participation with others outside of the site research team. The parent/guardian +/- participant will be given the opportunity to ask questions and if they agree to participation in the trial, they will be asked to sign and date the latest version of the Informed Consent Form (ICF). Similarly, there are two versions of the ICF: *version a* is for use at sites with liquid nitrogen available and *version b* for use at sites without liquid nitrogen availability. The parent/guardian must give explicit consent for the regulatory authorities, members of the research team and or representatives of the sponsor to be given direct access to the participant's medical records. In addition, a Child/Young Person Assent form will also be used for those aged 8 years and above, when appropriate.

The ICF contains optional items relating to consent for: the use of blood samples taken during the trial in future research; the taking, storage, analysis and use in future research of tissue biopsies (*version a* only); the taking, storage and genetic analysis of blood samples; the use of data held by central NHS bodies (see below); and the retention of contact information to enable future contact for consideration of participation in follow-up studies. The parent/guardian may provide or decline consent for any of these items independently.

The Principal Investigator or delegate will then sign and date the ICF +/- Assent form; a copy will be given to the parent/guardian, a copy will be filed in the medical notes, and the original placed in the Investigator Site File (ISF). Once the participant is entered into the trial, their trial number will be entered on the ICF maintained in the ISF. In addition, if the parent/guardian has given explicit consent, a copy of the signed ICF will be sent to the Birmingham Clinical Trials Unit (BCTU) trial team for review.

Details of the informed consent discussions should be recorded in the participant's medical notes in accordance with Good Clinical Practice (GCP). This should include date of discussion, the name of the trial, outcome of the discussion, version number of the PIS given to the family, version number of ICF signed and date consent received, and that the person signing the

consent form on behalf of the child has been determined to have parental responsibility. If a translator has been used this should be noted in the patient medical records. A copy of PIS should also be added to the medical notes.

Throughout the trial, the parent/guardian +/- participant will have the opportunity to ask questions about the trial. Any new information that may be relevant to their continued participation will be provided. Where new information becomes available which may affect their decision to continue, they will be given time to consider and if happy to continue will be re-consented. Re-consent will be documented in the medical notes. The parent/guardian's right to withdraw the participant from the trial will remain.

Electronic copies of the site-specific PIS and ICF will be available from the DESTINY Trial Office to be printed at the local site. Details of all parents/guardians approached about the trial will be recorded on a **DESTINY Participant Screening and Enrolment Log** and with the parent/guardian's prior consent, their General Practitioner (GP) will also be informed that the participant is taking part in the trial.

This study includes an optional consent to allow linkage to patient data available in NHS routine clinical datasets, such as the Paediatric Intensive Care Audit Network (PICANet), Hospital Episode Statistics (HES) and mortality data from the Office of National Statistics (ONS) through The Health and Social Care Information Centre and other central UK NHS bodies. The consent will also allow access to other new central UK NHS databases that may appear in the future. It will allow us to cross-check relevant outcomes against routine data sources and extend the follow-up of patients in the trial and collect long-term outcome and health resource usage data without needing further contact with the study participants. This is important as it will link treatments that may become a clinical standard of care to long-term outcomes that are routinely collected in clinical data, but which will not be collected during the follow-up period of the trial.

6 ENROLMENT AND RANDOMISATION

6.1 Enrolment and Screening

Patient eligibility will be confirmed by a member of the research team who is medically qualified, i.e. by a doctor. All baseline assessments will be carried out after informed consent and prior to patient randomisation.

At baseline, prior to randomisation, the following assessments will be performed:

- Patient demographics
- Preoperative weight and height/length
- Resting oxygen saturations
- Preoperative haematocrit
- Comorbidities
- Preoperative medications

6.2 Randomisation

6.2.1 Randomisation Methodology

Participants will be randomised by computer at the level of the individual in a 1:1 ratio to either del Nido cardioplegia or St. Thomas' blood cardioplegia.

A minimisation algorithm will be used within the online randomisation system to ensure balance in the treatment allocation over the following variables:

- Age: neonate (0-30 days), infant (31 days<1 year), child (1<7 years), older child (7<16 years)
- Incision or resection of ventricular myocardium anticipated (yes/no)
- Surgical centre

A 'random element' will be included in the minimisation algorithm, so that each participant has a probability (unspecified here), of being randomised to the opposite treatment that they would have otherwise received.

Full details of the randomisation specification will be stored in a confidential document at BCTU.

6.2.2 Blinding

As the technique for delivery of cardioplegia and interval between doses necessarily differs between treatment groups, patient safety may be compromised by blinding those administering the cardioplegia. The operating surgeon, perfusionist, anaesthetist, theatre scrub team and research nurse therefore will *not* be blinded to the intervention.

However, as the cardioplegia is only administered during surgery, patients, parents/guardians, and outcome assessors such as cardiologists, other surgeons, PICU medical and nursing staff, and ward staff will be blinded to the allocation. This will be maintained by only documenting the use of cardioplegia in the medical notes without referring to which product was used.

6.2.3 Unblinding

Adherence to blinding will be rigorously maintained by concealing the perfusion chart, containing the treatment allocation and details of its delivery. At sites with a paper perfusion chart, this will be placed in a sealed envelope within the medical notes *before* leaving theatre. At sites with an electronic perfusion chart, access to the data will be restricted to prevent inadvertent unblinding and views will be automatically logged. Allocation to the treatment will not be recorded in patient's electronic medical records. In the unlikely event of an emergency in which knowledge of the allocation may affect patient care or other safety reasons, the sealed envelope may be opened. Any member of staff accessing these documents will be required to record their name, sign, date/time, and the reason for opening the envelope, and each site will be monitored. At patient handover to PICU following surgery, information on the type, dosing, and timing of the cardioplegia will not be provided to the receiving team.

6.2.4 Randomisation Process

After participant eligibility has been confirmed and informed consent has been received, the participant can be randomised into the trial. Randomisation Notepads will be provided to investigators and will be used to collate the necessary information prior to randomisation. All questions and data items on the Randomisation Notepad must be answered before a Trial Number can be given. If data items are missing, randomisation will cease and must be re-started anew once the information is available.

Randomisation will be provided by a secure online randomisation system at the Birmingham Clinical Trials Unit (BCTU) (available at https://www.trials.bham.ac.uk/DESTINY). Unique log-in usernames and passwords will be provided to those authorised to use the online system and who have been delegated the role of randomising participants into the study as detailed on the **DESTINY Site Signature and Delegation Log**. These unique log-in details must not be shared with other staff and in no circumstances should staff at sites access either the randomisation process or trial database using another person's login details. The online randomisation system will be available 24 hours a day, 7 days a week, apart from short periods of scheduled maintenance

6.2.5 Randomisation Records

Following randomisation, a confirmatory e-mail will be sent to the randomiser (usually research nurse or perfusionist), DESTINY Trial Manager and CI.

Investigators will keep their own study file log which links participants with their allocated trial number in the **DESTINY Participant Recruitment and Identification Log**. The Investigator must maintain this document, which is **not** for submission to the Trials Office. The Investigator will also maintain the **DESTINY Participant Screening and Enrolment Log** which will be housed electronically on the REDCap database. The **DESTINY Participant Recruitment and Identification Log** and **DESTINY Participant Screening and Enrolment Log** should be held in strict confidence.

6.2.6 Informing Other Parties

The participant's General Practitioner will be notified that they are taking part in the trial using the **DESTINY GP Letter**. Allocation to the trial treatment will not be disclosed to GP. No other parties will be specifically informed of participation.

7 TRIAL TREATMENT / INTERVENTION

7.1 Intervention(s) and Schedule

The trial interventions are either del Nido cardioplegia (experimental arm) or St. Thomas' blood cardioplegia (control arm). The two cardioplegia solutions are compared in ***Table 1***.

del Nido cardioplegia	St. Thomas' blood cardioplegia (Harefield preparation)
1 litre Plasma-Lyte A base solution to which the following are added:	
Mannitol 20%, 16.3ml	Sodium chloride 8.6g
Magnesium sulfate 50%, 4ml	Magnesium chloride 6.262g
Sodium bicarbonate 8.4%, 13ml	Calcium chloride 330mg
Potassium chloride 2mEq/ml, 13ml	Potassium chloride 6.252g
Lidocaine 1%, 13ml	Procaine hydrochloride 1364mg
Total volume: 1052.8ml	In water for injection, per 1000ml

Table 1. *Table comparing the crystalloid components of the experimental (del Nido) and control (St. Thomas') treatments [42].*

The del Nido and St. Thomas' blood cardioplegia also differ in their formulations and doses as depicted in ***Table 2***.

Arm	IMPs	Formulations	Dose
Experimental	del Nido cardioplegia	1:4 blood:crystalloid preparation	Initial dose of 20ml/kg and subsequent doses every 60-90 minutes if required, at the discretion of the surgeon
Control	St Thomas' blood cardioplegia	4:1 blood:crystalloid using Harefield Hospital preparation	Initial dose of 20-30ml/kg, subsequent doses of 15 ml/kg every 20-30 minutes, at the discretion of the surgeon

Table 2. *Acceptable formulations of IMP and dosage of experimental vs control arm.*

The allocated cardioplegia solution will be administered according to the above preparation and schedule by the Clinical Perfusionist, at the request of the operating surgeon, in an antegrade manner via an aortic root or direct coronary ostia canula, following placement of a cross-clamp on the proximal aorta. The glass bottle containing the IMP should be vented using a spike vent or similar during infusion. By placing a waste sucker into the right atrium after commencing its delivery, the systemic bioavailability of the cardioplegia constituents following passage through the coronary circulation will be markedly reduced.

7.2 Drug Interaction or Contraindications

Other than the use of an alternative cardioplegia solution for the index operation, there are no restrictions on permitted, prohibited or concomitant medications in this trial. The exclusion criteria include known hypersensitivity or allergy to lidocaine (del Nido cardioplegia) or procaine (St. Thomas' blood cardioplegia).

7.3 Treatment Modification

In patients who require repeat aortic cross-clamping during the index procedure (predicted to be <5%), the same cardioplegia solution should be used, if required, but the dose given will be at the discretion of the operating surgeon, i.e. a smaller dose may be considered for an anticipated short period of repeat cross-clamping and recorded in the Perfusion CRF.

In those who require an unplanned reoperation or planned procedure requiring cardioplegia within 30 days of the index procedure, the standard cardioplegia for the centre should be used rather than the IMP. Additional use of cardioplegia during an unplanned procedure within the follow-up period should be recorded in the **Unplanned Reoperation/Intervention CRF**.

7.4 Cessation of Treatment / Continuation after the Trial

Cardioplegia is only given during planned surgical ischaemia and therefore the trial treatment will cease once the aortic cross-clamp is removed during the index procedure; if more than one aortic cross-clamping is required, this will be when it is removed for the final time.

7.5 Treatment Supply and Storage

7.5.1 Treatment Supplies

Both del Nido and St Thomas' cardioplegia will be manufactured and distributed by Stockport Pharmaceuticals, an NHS pharmaceutical manufacturer specialising in sterile IMPs for clinical trials (MIA(IMP) 13523). Drug distribution will be via suitable temperature-controlled processes.

On receipt of a batch of either cardioplegia solution, the NHS recipient must carry out routine checks to ensure that the drug has arrived in a way that is consistent with its despatch i.e. no visible particulate matter, discolouration, leakage, or evidence of temperature deviation. Where any issues are identified, such stock should be quarantined until guidance from the DESTINY Trial Office is received as to whether drug can be used or should be returned to the supplier.

7.5.2 Packaging and Labelling

Stockport Pharmaceuticals will package and label the IMPs in accordance with Annex 13 of the Clinical Trials Directive and as per the MHRA approved template, as below:

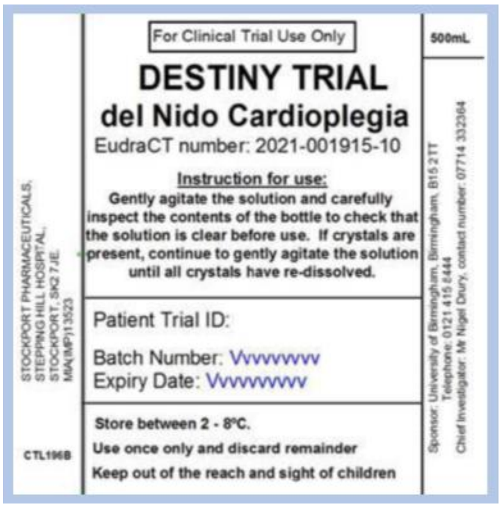

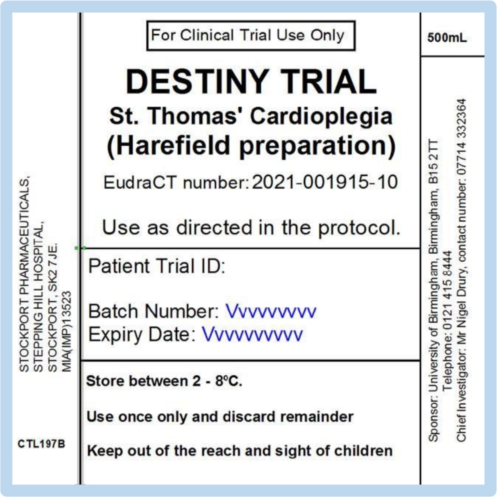

7.5.3 Drug Storage

Upon arrival at site pharmacy from Stockport Pharmaceuticals, the cardioplegia solutions will be stored in a suitable fridge with temperature monitoring capabilities at 2-8°C and segregated from routine clinical stock. Any temperature deviations should be reported to the DESTINY Trial Office using the **DESTINY Temperature Deviation Log** immediately upon becoming aware and stock should be quarantined until further guidance becomes available.

Batches of the cardioplegia will be despatched from the hospital pharmacy to the operating theatre for further storage in a new, dedicated, temperature-monitored refrigerator at 2-8°C and in the original packaging, segregated from routine clinical stock with access restricted to perfusion staff only.

7.6 Accountability and Compliance Procedures

7.6.1 Compliance

The drug is administered by the perfusionist during the index surgery according to unit practice and compliance can therefore be verified by the Perfusion CRF.

7.6.2 Accountability

Responsible site pharmacy personnel must maintain accurate accountability records of both cardioplegia solutions, including, but not limited to, the number of bottles received, the number of bottles transferred to theatres, batch number, expiry date, and date of transfer. The batch number of each bottle administered will be recorded on the Perfusion CRF. Used bottles of both cardioplegia solutions should be disposed of according to normal local procedures.

DESTINY Accountability Logs will be provided by the DESTINY Trial Office for the site pharmacy and theatres to log receipt of batches containing individual bottles of cardioplegia. “Use” includes disposal or destruction of bottles of cardioplegia that have expired or not been used for any reason, including breakage, discolouration, particulate matter, or temperature deviation. Disposal and destruction should be achieved using local protocols.

8 OUTCOME MEASURES AND STUDY PROCEDURES

8.1 Primary Outcome Measure

Area under the time-concentration curve (AUC) for plasma high-sensitivity troponin-I (µg.h/L) in the first 24 hours after the index aortic cross-clamp release (reperfusion).

8.2 Secondary Outcome Measures

- Low cardiac output syndrome (LCOS) defined as either of the following in the first 48 hours after reperfusion: Vasoactive Inotrope Score (VIS) ≥15 [1,2], or major cardiac event (cardiac arrest, ECLS or death) (n) [3]

- Duration of mechanical ventilation (hours), defined as the number of hours from termination of index CPB to extubation

- Length of postoperative stay on Paediatric Intensive Care (hours), defined as number of hours from admission to PICU from theatre following index procedure to discharge from PICU

- Maximum VIS by thresholds: ≥10, ≥15 and ≥20 in the first 48 hours (n)

- Total VIS in the first 4 hours after PICU admission following the index procedure (score)

- Arterial lactate (mmol/L) in the first 12 hours

- *Omega*, determined by [SaO_2]/[SaO_2-$ScvO_2$] in the first 12 hours [4]

- Total aortic cross-clamp time (mins)

- Total volume of cardioplegia given (ml)

- Need for internal defibrillation during reperfusion (n)

- Delayed sternal closure, incidence (n) and duration (days)

- Unplanned reoperation, including chest re-opening on PICU (n)

- Need for new renal replacement therapy (n)

- Lowest estimated glomerular filtration rate (eGFR), calculated using the bedside Schwartz equation and the peak postoperative creatinine on routine monitoring during the first 7 days following the index procedure (ml/min/1.73m^2), and according to the paediatric RIFLE categories (n) [5]

- Length of postoperative stay in the hospital (days), defined as number of days from day of surgery to discharge from hospital or death, whichever is sooner

- 30-day survival (n)

Detailed information on postoperative healthcare resource use will be collected on all patients from the Paediatric Critical Care Minimum Data Set (PCCMDS) [43], via the Paediatric Intensive Care Audit Network (PICANet), to which all centres routinely contribute. This dataset will be compared with data collected on the trial CRFs to assess the feasibility of using routinely collected outcome data in a trial setting and to inform planning of cost-effectiveness analyses in future trials.

8.3 Study procedures

Blood samples for troponin analysis: Blood will be drawn from the indwelling arterial or central venous line at each of the predetermined timepoints: baseline (after induction of anaesthesia but prior to sternotomy) and at 3 hours, 6 hours, 9 hours, 12 hours, and 24 hours after reperfusion. If there is more than one period of aortic cross-clamping during the index operation, times should be calculated from the first cross-clamp removal. Each sample will be approximately 2ml, so in total approximately 12ml will be taken for troponin analysis. Plasma samples will be collected in EDTA tubes, spun, and split into two aliquots of at least 400 microL; if samples are taken overnight, they may be stored in a specified location in a PICU refrigerator and processed the following morning. Aliquots will be stored in a remotely monitored freezer at -80°C at the local site until transfer to Birmingham City Hospital, Birmingham or Midland Metropolitan University Hospital, Smethwick, for analysis of hs-troponin-I (Abbott, Abbott Park, IL). Samples will be analysed in batches approx. every 6 months so that data on the primary outcome is available to the DMC prior to each scheduled meeting. Additional details will be provided in the **DESTINY Troponin samples SOP**.

Blood gas samples: Arterial lactate and arterial/central venous oxygen saturations (for calculation of *omega*) will be obtained from analysis of routine blood gas samples obtained at baseline (after induction of anaesthesia but prior to sternotomy) and at 3 hours, 6 hours, 9 hours, and 12 hours after reperfusion. If there is more than one period of aortic cross-clamping during the index operation, the times should be calculated from the first cross-clamp removal

Blood sample for genetic analysis: With specific consent, 6ml of blood will be drawn from the indwelling arterial line at baseline into an EDTA tube and transferred to the local site laboratory. The buffy coat will be isolated, decanted into a standard 1.5ml Eppendorf tube, or similar, and stored in a remotely monitored freezer at -80°C until transfer in batches to the University of Birmingham where DNA extraction will be performed. Further details will be provided to each site in the **DESTINY Genetic samples SOP**. At present, the extracted DNA will remain stored at -80°C and if additional funding is secured, genomic analysis will be performed.

Biopsy samples for metabolic analysis: At sites with liquid nitrogen available and with specific consent, myocardial tissue will be obtained from the right atrium during its routine incision at the onset of ischaemia and again at the end of ischaemia, in all patients. In patients undergoing routine resection of ventricular myocardium, such as hypertrophic septoparietal trabeculae from the right ventricular outflow tract or right ventricular free wall for anastomosis of an RV-PA conduit, these samples will be obtained during ischaemia when routinely resected. Specimens will each be washed in ice cold normal saline, placed in a cryotube (supplied), promptly snap-frozen in liquid nitrogen and stored in a remotely monitored freezer at -80°C at the local site until transfer for analysis at the University of Liverpool. Additional details will be provided to each site in the **DESTINY Biopsy samples SOP**.

Sample retention at the end of the trial: It is expected that the process of analysis will result in the destruction of all blood and tissue samples analysed. At the end of the trial, application(s) may be made for any remaining samples, that have not been analysed within the scope of the main trial or sub-studies, to be used in future ethically approved research. Otherwise, all remaining samples will be either destroyed or transferred to the Human Biomaterials Resource Centre at the University of Birmingham.

8.4 Schedule of Assessments

***Table** 3* below shows data collected at defined time points during the trial.

	Pre-operative	Pre-sternotomy	Intraoperative			Before leaving theatre	On PICU admission	Time since aortic cross-clamp removal (hours)						Daily until discharge	Hospital discharge
			Onset ischaemia	During ischaemia	Late ischaemia			3	6	9	12	24	48		
Screening	x														
Consent	x														
Randomisation	x														
Clinical baseline data[1]	x														
Blood for hs-troponin-I		x						x	x	x	x	x			
Arterial and central venous blood gases		x						x	x	x	x				
Administer IMP or control cardioplegia			x												
Intraoperative biopsies[2]			x	x	x										
Conceal perfusion chart						x									
Inotrope data							x	x	x	x	x	x	x		
Other clinical outcome data[3]							x				x	x	x	x	x
SAE reporting if required												x	x	x	x

***Table 3**. Schedule of assessments.*

[1] As collected in the Baseline CRF and described in section 8.2

[2] Right atrial +/- right ventricular biopsies at centres with liquid nitrogen available, as per section 8.3.

[3] As collected in the Postoperative CRF

8.5 Participant Withdrawal and Changes of Status Within Trial

Informed consent is defined as the process of learning the key facts about a clinical trial before deciding whether to participate.

Parents/guardians should be aware at the beginning of the process that they can freely withdraw (discontinue participation of) the participant from the trial at any time. A parent/guardian who withdraws their child from the trial does so completely (i.e. from trial treatment and all follow up) and is not willing to have any of their subsequent data used in any future trial analysis. If a child is randomised but subsequently withdrawn prior to surgery, the child will not receive the trial intervention and therefore there will be no outcome data to be collected for them. However, where appropriate, these patients will still be reported in the formal analyses, such as in the consort flow diagram.

A parent/guardian who wishes to end their child's participation in a particular aspect of the trial, will be considered as having changed their status within the trial.

The changes in status within the trial are categorised in the following ways:

- No trial related follow-up: The parent/guardian would no longer like the participant to undergo trial assessments in accordance with the protocol schedule but is willing for routine data to be collected from standard follow-up and if applicable using any central UK NHS bodies for long-term outcomes (i.e. the participant's data can be collected at standard assessments and used in the trial analysis, including data collected as part of long-term outcomes).

- No biopsy sub-study: The parent/guardian would no longer like the participant to take part in the biopsy sub-study. If this occurs prior to surgery, no study biopsies will be taken during the procedure; if it occurs after surgery, the investigator will confirm whether any biopsies already obtained can be kept and analysed.

- No genetic sub-study: The parent/guardian would no longer like the participant to take part in the genetic sub-study. If this occurs prior to surgery, no blood sample for genetic analysis will be taken prior to the procedure; if it occurs after surgery, the investigator will confirm whether any blood already obtained can be kept and analysed.

A patient may also be withdrawn from the trial prior to their index operation for clinical reasons e.g. change of operation not requiring cardioplegia. The details of either withdrawal or change of status within the trial (date, reason, and category of status change) should be clearly documented in the source data and on the Trial Exit/Change of Status CRF .

Patients found to be ineligible post randomisation should be followed up according to all trial processes and will still have their data analysed unless they explicitly change their level of participation.

9 ADVERSE EVENT REPORTING

9.1 Definitions

The ***Table 4*** shows adverse event classification:

Adverse Event	**AE**	Any untoward medical occurrence in a participant or clinical trial subject administered a medicinal product and which does not necessarily have a causal relationship with this treatment.
Adverse Reaction	**AR**	All untoward and unintended responses to an IMP related to any dose administered.
Serious Adverse Event	**SAE**	Any untoward medical occurrence or effect that:<br>• Results in death or is life-threatening[1]<br>• Requires hospitalisation or prolongation of existing hospitalisation<br>• Results in persistent or significant disability or incapacity<br>• Is a congenital anomaly/birth defect<br>• Or is otherwise considered medically significant by the Investigator[2]
Serious Adverse Reaction	**SAR**	An Adverse Reaction which also meets the definition of a Serious Adverse Event
Unexpected Adverse Reaction	**UAR**	An AR, the nature or severity of which is not consistent with the applicable product information (e.g. Investigator Brochure for an unapproved IMP or (compendium of) Summary of Product Characteristics (SPC) for a licensed product).<br>When the outcome of an AR is not consistent with the applicable product information the AR should be considered unexpected.
Suspected Unexpected Serious Adverse Reaction	**SUSAR**	A SAR that is unexpected i.e. the nature, or severity of the event is not consistent with the applicable product information. A SUSAR should meet the definition of an AR, UAR and SAR.

Table 4*. Definitions of types of adverse events.*

[1] *The term life-threatening is defined as diseases or conditions where the likelihood of death is high unless the course of the disease is interrupted.*

[2] *Medical events that may not be immediately life-threatening or result in death or hospitalisation but may jeopardise the patient or may require intervention to prevent one of the other outcomes listed in the definitions above.*

9.2 Adverse Events - General Recording Requirements

The collection and reporting of Adverse Events (AEs) will be in accordance with the UK Policy Framework for Health & Social Care (2017), the requirements of the Health Research Authority (HRA), and the sponsor's Code of Practice for Research. Definitions of the different types of AEs are listed in table of definitions, section 9.1.

It is routine practice to record adverse events in the patient's medical notes and it is also recommended that this includes the documentation of the assessment of severity and seriousness, and for causality (relatedness) in relation to the intervention(s) in accordance with the protocol. The assessment of causality should be made with regards to the Reference Safety Information (RSI). For the purposes of the DESTINY trial, this will be the Investigator Brochure for del Nido cardioplegia and the Summary of Product Characteristics for St Thomas' cardioplegia.

9.3 Adverse Event Reporting in DESTINY

The safety profile for this trial population and interventions are well established so although it is recommended that the severity, seriousness, and causality of all AEs should be recorded in the source data, a strategy of targeted reporting of AEs will therefore not affect the safety of participants. The reporting of only a subset of AEs via the Postoperative CRF, from the commencement of protocol defined treatment until 30 days, is consistent with aims of the trial.

9.4 Serious Adverse Event (SAE) Reporting in DESTINY

SAE reporting using standard definitions and practices for adult drug trials are not appropriate for clinical trials involving cardiac surgery in children [44] as events typically classified as SAEs occur commonly in the usual perioperative course for these children; their use would lead to a significant reporting burden and may detract from those events which are considered critical. A recent multi-centre UK study has defined important early morbidities associated with paediatric cardiac surgery as part of a wider study on morbidity in these patients [3], which have been adopted by the UK National Congenital Heart Disease Audit [45]. We therefore will use or adapt these definitions of important morbidities as the basis for expedited reporting of SAEs in this trial of children of all ages undergoing non-emergency cardiac surgery.

As the period of data collection lasts for 30 days, participants will remain under follow-up at the same hospital for the duration of their participation in the trial such that the local research team will collect all trial data on all events meeting the definition of SAEs directly. If the patient has been discharged home within 30 days, the occurrence of any SAE within this timeframe will be ascertained either during a routine follow-up clinic visit or by a telephone call.

9.5 SAEs not requiring expedited reporting to BCTU

Where the safety profile is well established, the causal relationship between either the intervention (or the participant's condition), and the SAE, may be known. That is, such events are protocol-defined as 'expected', will be considered as complications rather than adverse events if they occur within 30 days of the index operation, and will be recorded on the Postoperative CRF. This list is categorised by organ system, adapted from that used in the Pediatric Heart Network's Single Ventricle Reconstruction trial [46] and informed by the definitions developed during the Cardiac Morbidity study [3].

Cardiovascular

- Arrhythmia, requiring medication or other treatment, excluding electrolyte supplementation
- Impaired cardiac function: Vasoactive Inotrope Score ≥15 at 48 hours after the index operation [1]

- Repeat aortic cross-clamp, unplanned during index operation
- Pericardial effusion or collection, requiring drainage
- Superior or inferior vena caval obstruction
- Other thrombosis or thromboembolism, demonstrated on imaging
- Other cardiovascular not requiring expedited reporting

Respiratory

- Chylothorax requiring intervention, whether drainage, dietary or medication
- Haemothorax, pleural effusion or pneumothorax, requiring drainage
- Hypoxaemia, requiring unplanned escalation of care
- Intubation for >2 weeks after the index operation
- Prolonged pleural effusion, requiring drainage for >10 days after the index operation
- Reintubation, any cause
- Other respiratory, including tracheal injury, vocal cord injury, or airway obstruction requiring intervention

Neurological

- Any new neurological deficit persisting at hospital discharge
- Other neurological not requiring expedited reporting

Renal

- Renal replacement therapy: peritoneal dialysis or haemofiltration for renal failure (oligo-anuria of <0.5ml/kg/hr and elevated creatinine for age) and/or fluid overload, initiated as a new support within 30 days after the index operation. Excludes renal support on ECLS [3].

Gastrointestinal

- Gastrointestinal bleed, requiring treatment
- Liver failure, defined as INR ≥1.5 not corrected by parenteral administration of vitamin K [47], or AST or ALT ≥950 IU/L [48]
- Necrotising enterocolitis: systemic and abdominal signs consistent with necrotising enterocolitis, with or without radiological signs, and commenced on treatment by a paediatric surgery specialist [3].
- Oesophageal or bowel perforation, not associated with necrotising enterocolitis
- Other gastrointestinal

Infection

- Blood stream infection, including both catheter and non-catheter related, with systemic signs of infection, a positive culture not judged to be a contaminant, and for catheter-related, positive cultures from the line or line tip [3]
- Empyema
- Endocarditis, based on diagnostic clinical, imaging or culture evidence
- Gastroenteritis
- Pneumonia, requiring treatment
- Surgical site infection, superficial
- Urinary tract infection
- Other infection not requiring expedited reporting

Other complication

- Readmission to ICU, any cause
- Other not requiring expedited reporting

9.6 Events that require expedited reporting to the Sponsor on the SAE Form

Based on those morbidities identified as important in the recent multi-centre UK study [3], the following SAEs are protocol-defined as expected but must be reported in an expedited manner using the Serious Adverse Event reporting form to enable efficient monitoring of frequency of events; this requirement has been agreed with the DMC prior to commencing recruitment:

- **Death**
- **Extracorporeal life support (ECLS)**: Use of extracorporeal support following the index operation, whether started in the operating theatre or in the ICU, and whether the indication was cardiac arrest, low cardiac output state, poor cardiac function, arrhythmia, residual or recurrent cardiac lesion, or sepsis.
- **Major adverse cardiac event**: Includes cardiac arrest, where the child receives any chest compressions or defibrillation; chest reopening on the ICU or ward, for any reason; major haemorrhage in the ICU following surgery, defined as >10ml/kg/hr for 2 consecutive hours; and acute shunt failure, in those with a surgically constructed systemic to pulmonary shunt.
- **Unplanned reoperation/intervention in the early postoperative period**: Cardiac surgical operations with or without cardiopulmonary bypass, or interventional catheter procedures that occur within 30 days after the index operation or within the same hospital admission, not intended during the planning phase. Includes diaphragm plication and pacemaker insertion for surgically acquired arrhythmias but excludes planned diagnostic or interventional cardiac catheterisation, delayed chest closure, procedures for bleeding, institution of or weaning from ECLS, and non-cardiac surgical operations.
- **Acute neurological event**: New abnormality detected on clinical evaluation: coma; intracranial haemorrhage or stroke (confirmed on imaging); seizures (confirmed on electroencephalogram or obvious motor signs); or other neurological injury likely to result in a persistent or permanent deficit.
- **Sepsis**: defined broadly as severe infection leading to life-threatening organ dysfunction
- **Deep surgical site infection**: Surgical site infection and/or mediastinitis including any infection of an incised wound that undergoes any surgical reintervention within 30 days after the index operation or within the same hospital admission, independent of culture positivity.
- **Other severe or life-threatening unexpected event**: Any other events which are severe or life-threatening must be formally assessed for relatedness (PI) and expectedness (CI).

If a patient fulfils more than one of the above SAE criteria, e.g. cardiac arrest requiring ECLS, a separate SAE form should be completed for each of the events.

The following events were classified as important morbidities but will not require expedited reporting: blood stream infection or endocarditis; feeding problems; necrotising enterocolitis; need for renal replacement therapy; and prolonged pleural effusion or chylothorax [3].

9.7 Reporting procedure

9.7.1 Reporting procedure for expedited SAEs by sites

On becoming aware that a participant has experienced an expedited SAE, the Investigator or suitably medically qualified delegate should report it to their own Trust in accordance with local practice and to the BCTU trials office as per section 9.6 above.

To report an expedited SAE to the BCTU trials office, the Investigator or delegate must complete, date, and sign the trial-specific SAE form. The completed form together with any other relevant, appropriately anonymised data should be scanned and sent as email attachments to destiny@trials.bham.ac.uk no later than 24 hours after first becoming aware of the event:

Where an SAE Form has been completed by someone other than the Investigator or delegate, initially, the original SAE form will need to be countersigned by the Investigator to confirm agreement with the causality and severity assessments.

On receipt of an SAE form, the BCTU trials team will transcribe the data onto the trial database. This results in a unique reference number being generated for the SAE, which the BCTU trials team will share with the site via email as proof of receipt of the SAE form. The site and the BCTU trials team should ensure that the SAE reference number is quoted on all correspondence and follow-up reports regarding the SAE and filed with the SAE in the Site File.

If the site has not received confirmation of receipt of the SAE from BCTU or if the SAE has not been assigned a unique SAE identification number within 1 working day, the site should contact the BCTU trials team.

9.7.2 Provision of follow-up information

Following the reporting of an SAE for a participant, the participant should be followed up until resolution or stabilisation of the event. Follow-up information should be provided using the SAE reference number provided by the DESTINY trial team by completing follow-up SAE reports and sending via email to the trial office. Once the SAE has been resolved, all critical follow-up information has been received and the paperwork is complete, this should be filed in the Site File, whilst BCTU trials office will retain copies.

9.7.3 Assessment of relatedness

When completing the SAE form, the PI will be asked to define the causality (relatedness) and the severity of the AE. In defining the causality, the PI must consider if any concomitant events or medications may have contributed to the event and, where this is so, these events or medications should be reported on the SAE form. It is not necessary to report concomitant events or medications which do not contribute to the event. The assessment of relatedness is depicted in ***Table 5.***

**Category**	**Definition**	**Causality**
Definitely	There is clear evidence to suggest a causal relationship, and other possible contributing factors can be ruled out.	Related
Probably	There is evidence to suggest a causal relationship, and the influence of other factors is unlikely.	
Possibly	There is some evidence to suggest a causal relationship. However, the influence of other factors may have contributed to the event (e.g. the participant's clinical condition, other concomitant events, or medication)	
Unlikely	There is little evidence to suggest there is a causal relationship. There is another reasonable explanation for the event (e.g. the participant's clinical condition, other concomitant events, or medication).	Unrelated
Not related	There is no evidence of any causal relationship.	

Table 5*. Assessment of relatedness.*

On receipt of an SAE Form, the Trials Office will forward it, with the unique reference number, to the Chief Investigator (CI) who will independently review the causality of the SAE. An SAE judged by the PI or CI to have a reasonable causal relationship with the intervention will be regarded as a related SAE (SAR). The causality assessment given by the PI will not be downgraded by the CI. If the CI disagrees with the PIs causality assessment, the opinion of both parties will be documented, and where the event requires further reporting, the opinion will be provided with the report. The CI will send their assessment of causality and, where applicable, expectedness (see 9.8 below) to the Trials Office.

9.8 Assessment of Expectedness by the CI

The CI will also assess all related SAEs for expectedness with reference to the following criteria (***Table 6***).

Category	Definition
Expected	An adverse event that is consistent with known information about the trial related procedures or that is clearly defined in the relevant safety information: DESTINY Investigator Brochure del Nido (v1.0, 30/03/2021) and SmPC Martindale Cardioplegia Concentrate (18/02/2015).
Unexpected	An adverse event that is not consistent with known information about the trial related procedures.

Table 6. *Assessment of expectedness.*

The CI will not overrule the severity or causality assessment given by the site PI but may add additional comment on these. If the event is unexpected (i.e. is not defined in the protocol as

an expected event or is not defined in the approved version of the RSI, it will be classified a Suspected Unexpected Serious Adverse Reaction (SUSAR).

9.9 Protocol defined expected SAEs

Cardiac surgery with cardioplegic arrest in children may be associated with many anticipated serious adverse events in the early postoperative period, affecting one or more organ systems. We have defined above in section 9.6 which SAEs require expedited reporting to the sponsor.

9.10 Reporting SAEs to third parties

9.10.1 Reporting to the Competent Authority and main Research Ethics Committee

A minimal data set of all individual events categorised as a fatal or life threatening SUSAR to the Medicines and Healthcare products Regulatory Agency (MHRA), main REC and Research Governance Team (RGT) by BCTU within 7 days. Detailed follow-up information will be provided within an additional 8 days. All other events categorised as non-life threatening SUSARs will be reported within 15 days.

9.10.2 Reporting to the Data Monitoring Committee

The Chair of the independent DMC will be notified of any death or life threatening SUSAR by BCTU within 7 days. All other SAEs requiring expedited reporting to the Sponsor will be reviewed at the subsequent DMC meeting. Details of all SUSARs and any other safety issue which arises during the trial will be reported to PIs. A copy of any such correspondence will be filed in the site file and TMF.

9.11 Urgent Safety Measures

If any urgent safety measures are taken, BCTU shall immediately, and in any event no later than 3 days from the date the measures are taken, give written notice to the REC and MHRA of the measures taken and the circumstances giving rise to those measures.

9.12 Monitoring Pregnancies for Potential Serious Adverse Events

Monitoring pregnancies is not applicable for this trial. All female patients of child-bearing age undergo pregnancy testing on admission prior to surgery; if positive, the patient would not usually proceed with surgery other than in an emergency, which is an exclusion for this trial.

10 DATA HANDLING AND RECORD KEEPING

10.1 Source Data

Source data is defined as all information in original records and certified copies of original records of clinical findings, observations, or other activities in a clinical trial necessary for the reconstruction and evaluation of the trial. To allow for the accurate reconstruction of the trial and clinical management of the subject, source data will be accessible and maintained from different sources (***Table 7).***

Data	Source
**Lab results**	The original lab report (which may be electronic) is the source data and will be kept and maintained, in line with normal local practice. Information will be transcribed onto CRFs.
**Imaging**	The source is the original imaging usually as an electronic file. Data may be supplied to the Trials Office as a password-protected, anonymised, copy of the electronic file, or as an interpretation of the imaging provided on a CRF. Where data is interpreted, the CRF onto which it is transcribed becomes the source. Copy of the CRF should be provided to the Trials Office
**Clinical event data**	The original clinical annotation is the source data. This may be found on clinical correspondence, or electronic or paper participant records.
**Trial-specific event data**	The main trial CRFs are the source for data that would otherwise not routinely be recorded in the patient's medical record e.g. whether intraoperative biopsies were obtained, and time taken.
**Recruitment**	The original record of the randomisation is the source. It is held on BCTU servers as part of the randomisation and data entry system.
**Drop out**	Where a participant expresses a wish to withdraw, the conversation must be recorded in the medical record.

Table 7. *Source data.*

10.2 Case Report Form (CRF) Completion

A CRF is required and should be completed for each individual subject. The data held on the completed original CRFs are the sole property of the respective PIs whilst the data set as a whole is the property of the Sponsor and should not be made available in any form to third parties, except for authorised representatives or appropriate regulatory authorities, without written permission from the sponsor. Appropriate data sharing requests will be considered by the Sponsor.

It will be the responsibility of the investigator to ensure the accuracy of all data entered in the CRFs and confirm accordingly. The **DESTINY Site Signature and Delegation Log** will identify all those personnel with responsibilities for data collection.

The CRFs will comprise the following forms:

Form Name	Schedule for submission
**Randomisation CRF**	At the point of randomisation
**Baseline CRF**	At the point of undergoing the index operation
**Operation CRF**	At the point of undergoing the index operation
**Perfusion CRF**	At the point of undergoing the index operation
**Supplementary Perfusion CRF**	At the point of undergoing the index operation (if required)
**Postoperative CRF**	At the point of hospital discharge
**Unplanned Reoperation/ Intervention CRF**	At the point of undergoing each episode of further unplanned surgery or catheter intervention
**Serious Adverse Event CRF**	Emailed within 24 hours of research staff at site becoming aware of an SAE requiring expedited reporting
**Supplementary Serious Adverse Event CRF**	At the point of submitting a Serious Adverse Event CRF with additional medications (if required)
**Exit/Change of status CRF**	At the point of change of status, withdrawal from the trial or death

Data should be submitted in a timely manner, therefore if data has not been provided within four weeks of the submission schedule detailed **in *Table 8.*** *Trial Case report forms*

Data reported on each form will be consistent with the source data and any discrepancies will be explained. Data should be submitted in a timely manner, therefore if data has not been provided within four weeks of the submission schedule detailed in *Table 8*, then a reminder email will be sent to sites. If the data has still not been received within 6 weeks, then the trial manager will directly contact the site via telephone to ascertain the reason for the delay. At 8 weeks from expected submission if the data still has not been received this may be escalated to site's senior management and can trigger a monitoring visit.

All missing and ambiguous data will be queried as per section 10.3.2. Staff delegated to complete CRFs will be trained to adhere to the **DESTINY CRF Completion Guideline**.

The following guidance applies to data and partial data:

- Date format: all dates should be recorded as dd-mmm-yyyy
- Time format and unknown times: all times should be in accordance with the 24hr clock
- Trial-specific interpretation of data fields: where guidance is needed additional information will be supplied
- Entry requirements for concomitant medications (generic or brand names): generic names should be used where possible
- Missing/incomplete data: should be clearly indicated, all blank fields will be queried by the trial office
- Repeat laboratory tests: the data used to inform clinical decisions should always be supplied. If a test is repeated, it is either to confirm or clarify a previous reading. Confirmatory tests should use the original test values. Protocol and GCP non-compliances should be reported to the Trials Office on discovery.

In all cases it remains the responsibility of the site PI to ensure that the CRF has been completed correctly and that data are accurate. This will be evidenced by the electronic signature, accessed by unique login, of the site PI on the CRF. Only CRFs specified in the protocol must be used.

10.3 Data Management

Processes will be employed to facilitate the accuracy of the data included in the final report. These processes will be detailed in the trial specific Data Management Plan. Coding and validation will be agreed between the trial team and the trial database will be signed off once the implementation of these has been assured.

For the DESTINY trial, CRFs will be an electronic record completed at site (except for Serious Adverse events which will be paper), only by those at site delegated the task of doing so. Forms will be considered "complete" once all data fields have been either completed unambiguously or it has been made explicit that the data is unobtainable. In all cases it remains the responsibility of the site's PI to ensure that the CRF has been completed correctly and that the data are accurate. This will be evidenced by the electronic signature of the site's PI on the CRF.

10.3.1 Source Data

The local trial team can collate data to be entered onto the electronic database using paper copies of the data forms as worksheets, for simplicity. Where data exists in written form prior to this collation, the original record is the source data. If the data is written directly onto the worksheet, without any previous written record, the worksheet itself becomes the source data. The local team need to have a consistent approach to the use of worksheets so that it is clear if they are to be considered source data or not.

Source data may be checked against the CRFs where on-site monitoring is conducted and must be available for verification.

10.3.2 Data Clarification

Missing and ambiguous data will be queried using a Data Clarification system in line with the **DESTINY Data Management Plan** and will focus on data required for trial outcome analysis and safety reporting. Single data entry with central monitoring will be employed. Staff at site (as delegated on the **DESTINY Site Signature and Delegation Log**) will enter and submit data, with the exception of serious adverse events, using an online electronic CRF at https://www.trials.bham.ac.uk/DESTINY. Unique log-in usernames and passwords will be provided to those who wish to use the online system and who have been delegated the role of CRF completion as detailed on the **DESTINY Site Signature and Delegation Log**. These unique log-in details must not be shared with other staff and in no circumstances should staff at sites access the trial database using another person's login details. The trial office will be unable to edit data forms entered by site staff. The system will include data validations to improve data quality (e.g. to prevent nonsensical dates or numerical values). Changes to the data on the system will be documented and attributable, with a reason for the change documented and will be made by local site staff (except for serious adverse events).

SAE Forms will be emailed directly to the trial office for trial office staff to enter the data on the electronic CRF online. Site staff will be unable to edit this data.

10.3.3 Self-evident corrections

No self-evident corrections will be made to the data.

.

10.4 Data Security

The security of the System is governed by the policies of the University of Birmingham. The University's Data Protection Policy and the Conditions of Use of Computing and Network Facilities set out the security arrangements under which sensitive data should be processed and stored. All studies at the University of Birmingham must be registered with the Data Protection Officer and data held in accordance with the Data Protection Act (2018) and subsequent amendments. The Study Centre has arrangements in place for the secure storage and processing of the study data which comply with the University of Birmingham policies.

The System incorporates the following security countermeasures:

- Physical security measures: restricted access to the building, supervised onsite repairs and storages of back-up tapes/disks in a fire-proof safe.
- Logical measures for access control and privilege management: including restricted accessibility, access-controlled servers, separate storage of non-identifiable data etc.
- Network security measures: including site firewalls, antivirus software, and separate secure network protected hosting etc.
- System Management: the system will be developed by the BCTU Programming Team and will be implemented and maintained by the BCTU Programming Team.
- System Design: the system shall comprise of a database and a data entry application with firewalls, restricted access, encryption and role-based security controls.
- Operational Processes: the data will be processed and stored within the Study Centre (University of Birmingham).
- Data processing: Statisticians will only analyse anonymised data.
- System Audit: The System shall benefit from the following internal/external audit arrangements:
    - Internal audit of the system
    - Periodic IT risk assessments
- Data Protection Registration: The University of Birmingham has Data Protection Registration to cover the purposes of analysis and for the classes of data requested. The University's Data Protection Registration number is Z6195856.

10.5 Archiving

The TMF will be stored at BCTU for at least 3 years after the end of the trial. Long-term offsite data archiving facilities will be considered for storage after this time; data will be stored securely and confidentially for at least 25 years. BCTU has standard processes for both hard copy and computer database legacy archiving.

It is the responsibility of the PI to ensure all essential trial documentation and source documents (e.g. signed ICFs, Investigator Site Files, Pharmacy Files, participants' hospital notes, copies of CRFs etc.) at their site are securely retained for at least 25 years.

11 QUALITY CONTROL AND QUALITY ASSURANCE

11.1 Site Set-up and Initiation

All PIs will be asked to sign the necessary agreements including a site signature & delegation log between the PI and CTU and supply a current CV and GCP certificate to BCTU. All site staff who are performing trial specific tasks are required to sign the **DESTINY Site Signature and Delegation Log**, which details which tasks have been delegated to them by the PI.

Prior to commencing recruitment, each recruiting site will undergo a process of initiation, either a meeting or a teleconference, at which key members of the site research team are required to attend, covering aspects of the trial design, protocol procedures, adverse event reporting, collection and reporting of data and record keeping. Sites will be provided with an Investigator Site File and a Pharmacy File containing essential documentation, instructions, and other documentation required for the conduct of the trial. The BCTU trials team must be informed immediately of any change in the site research team.

11.2 Monitoring

The monitoring requirements for this trial have been developed following trial specific risk assessment by BCTU and as documented in the monitoring plan.

11.3 Onsite Monitoring

The trial sites will be monitored in accordance with the **DESTINY Monitoring Plan**. Any monitoring activities will be reported to the trials team and any issues noted will be followed up to resolution. Additional on-site monitoring visits may be triggered, for example by poor CRF return, poor data quality, low SAE reporting rates, excessive number of participant withdrawals or deviations (also defined in the monitoring plan). Investigators will allow the DESTINY trial staff access to source documents as requested. The monitoring will be conducted by DESTINY trial manager or other suitable BCTU representative.

11.4 Central Monitoring

Trials staff will check incoming ICFs and CRFs for compliance with the protocol, data consistency, missing data and timing at a frequency and intensity determined by the Data Management Plan. Sites will be sent DCFs requesting missing data or clarification of inconsistencies or discrepancies.

Sites will be requested to send in copies of signed ICFs and other documentation for central review for all participants providing explicit consent. This will be detailed in the monitoring plan.

11.5 Audit and Inspection

The Investigator will permit trial-related monitoring, audits, ethical review, and regulatory inspection(s) at their site, providing direct access to source data & documents. The investigator will comply with these visits and any required follow up. Sites are also requested to notify BCTU of any relevant inspections.

11.6 Notification of Serious Breaches

Sites may be suspended from further recruitment in the event of serious and persistent non-compliance with the protocol and/or GCP, and/or poor recruitment. Any major problems identified may be reported to Trial Steering Committee and the REC. This includes reporting serious breaches of GCP and/or the trial protocol to the REC and MHRA.

In accordance with Regulation 29A of the Medicines for Human Use (Clinical Trials) Regulations 2004 and its amendments, the Sponsor of the trial is responsible for notifying the licensing authority in writing of any serious breach of the conditions and principles of GCP in connection with that trial or the protocol relating to that trial, within 7 days of becoming aware of that breach.

For the purposes of this regulation, a “serious breach” is a breach which is likely to affect:

- the safety or physical or mental integrity of the subjects of the trial
- the scientific value of the trial

Sites are therefore requested to notify the Trials office of any suspected trial-related serious breach of GCP and/or the trial protocol. Where the Trials Office is investigating whether a serious breach has occurred, sites are also requested to cooperate with the Trials Office in providing sufficient information to report the breach to the MHRA where required and in undertaking any corrective and/or preventive action.

12 END OF TRIAL DEFINITION

The end of trial will be 6 months after the last data capture, including resolution of data queries and completion of hs-troponin-I sample analysis. This will allow sufficient time for the completion of protocol procedures, data collection and data input. The BCTU trial team will notify the main REC, MHRA and RGT within 90 days of the end of trial. Where the trial has terminated early, the Trials Office will inform the MHRA and REC within 15 days of the end of trial. The Trials Office will provide them with a summary of the clinical trial report within 12 months of the end of trial.

A copy of the end of trial notification as well as the summary report will be sent to the MHRA and REC.

13 STATISTICAL CONSIDERATIONS

13.1 Sample Size

It is hypothesised that del Nido cardioplegia reduces myocardial injury during surgery compared with St. Thomas' blood cardioplegia. The DESTINY trial will use postoperative hs-troponin-I release to measure the level of myocardial injury which has been shown to strongly correlate with clinical outcomes including inotropic support, duration of ventilation, ventricular dysfunction, and early death [9,21].

The justification for the sample size is based on data from the BRICC trial. In a similar cohort of participants to those in the control group for this study, a mean postoperative hs-troponin release value of approximately 64.0 µg.h/L and a standard deviation of approximately 42.0 µg.h/L were observed.

To detect a difference of 30% (relative reduction, 19.2 µg.h/L absolute reduction) between groups using the standard method of a two-sample t-test and assuming equal variance with 90% power and a type I error rate of 0.05, 102 participants per group will need to be randomised, 204 in total. Assuming and adjusting for a 3% loss to follow-up/drop-out rate (a low rate of drop-out is expected as the primary outcome is measured at 24 hours and it is anticipated that all children undergoing heart surgery would still be in hospital at this point), 220 participants will need to be recruited.

13.2 Analysis of Outcome Measures

A separate Statistical Analysis Plan will be produced and will provide a more comprehensive description of the planned statistical analyses. A brief outline of these analyses is given below. The primary comparison groups will be composed of those treated with del Nido cardioplegia versus those treated with St. Thomas' blood cardioplegia. In the first instance, all analyses will be based on the intention to treat principle, i.e. all participants will be analysed in the treatment group to which they were randomised irrespective of compliance or other protocol deviation. For all outcome measures, appropriate summary statistics will be presented by group (e.g. proportions/percentages, mean/standard deviation, or median/interquartile range).

Intervention effects will be adjusted for the minimisation variables listed in section 6.2.1 where possible. No adjustment for multiple comparisons will be made.

13.2.1 Primary Outcome Measure

AUC for hs-troponin-I release in the first 24 hours (ng/L) will be calculated using the trapezoidal rule from samples taken at baseline, 3, 6, 9, 12 and 24 hours after aortic cross-clamp release.

Missing baseline troponin values will be imputed using the median value of the participant's treatment group. Due to the low variability of the baseline measurements, this should not impact on the validity of the results. To ensure that this is the case, the results from the primary analysis will be compared with that of the complete case, to determine that they are consistent with one another.

Adjusted mean differences along with 95% confidence intervals will be estimated using a linear regression model. Statistical significance of the treatment group parameter will be determined from the p-value generated by the model. If AUC for hs-troponin-I release in the first 24 hours (ng/L) is too skewed, the natural log transformation of the AUC will be used before performing linear regression and the results from this model will be interpreted in the original scale appropriately.

13.2.2 Secondary Outcome Measures

Continuous data items will be analysed using a linear regression model. Results will be presented as adjusted mean difference and 95% confidence intervals.

Continuous outcomes measured across more than 3 time points (e.g. arterial lactate and omega) will be analysed as repeated measures using linear mixed models using all available data. Baseline value of the measure (if available) and time by treatment interaction will be included with time as a continuous variable. Results will be presented as mean difference and 95% confidence intervals.

Time-to-event data outcomes (such as time from admission to PICU to discharge from PICU) will be analysed using the Cox regression model. Results will be presented as Hazard ratio and 95% confidence intervals. Kaplan Meier plots will also be presented with the log-rank test for visual interpretation.

13.2.3 Subgroup Analyses

Subgroup analyses on the primary outcome will be limited to the same variables used in the minimisation algorithm (see section 6.2.1). Tests for statistical heterogeneity (e.g. by including the treatment group by subgroup interaction parameter in the statistical model) will be performed prior to any examination of effect estimate within subgroups. The results of subgroup analyses will be treated with caution and will be used for the purposes of hypothesis generation only.

13.2.4 Missing Data and Sensitivity Analyses

Every attempt will be made to collect full follow-up data on all study participants; it is thus anticipated that missing data will be minimal, especially given the short duration of follow-up. Randomised participants who do not undergo surgery cannot contribute data to the primary outcome and will therefore be excluded from the primary analysis. Additional participants may be randomised to ensure the sample size is maintained. Participants who undergo surgery but have missing primary outcome data for troponin at one or more timepoints after aortic cross-clamp release will not be included in the primary analysis in the first instance. This presents a risk of bias, and sensitivity analyses may be undertaken to assess the possible impact of the risk. Full details of all sensitivity analyses will be included in the Statistical Analysis Plan.

13.3 Planned Interim Analysis

Interim analyses of safety and efficacy for presentation to the independent DMC will take place during the study. The committee will meet prior to study commencement to agree the manner and timing of such analyses but this is likely to include the analysis of the primary and major secondary outcomes and full assessment of safety (SAEs) at least at annual intervals. Criteria for stopping or modifying the study based on this information will be ratified by the DMC. Details of the agreed plan will be written into the Statistical Analysis Plan. Further details of DMC arrangements are given in section 14.5.

13.4 Planned Final Analyses

The primary analysis be performed according to the Statistical Analysis Plan and will include data items up to and including discharge and no further. Longer term data will form part of the follow-up imaging sub-study but will be analysed separately once participants have completed the corresponding assessments.

14 TRIAL ORGANISATIONAL STRUCTURE

14.1 Sponsor

The University of Birmingham is the Sponsor.

14.2 Coordinating Centre

BCTU is the Coordinating Centre. Delegation of tasks to the BCTU, from the Sponsor, are documented in the **DESTINY Clinical Trials Task Delegation Log**.

14.3 Trial Management Group

The Trial Management Group (membership detailed in the Administrative Information section above) will monitor all aspects of the conduct and progress of the trial, ensure that the protocol is adhered to and take appropriate action to safeguard participants and the quality of the trial itself. It will meet approximately monthly.

14.4 Trial Steering Committee

The role of the DESTINY Trial Steering Committee (TSC) is to provide overall supervision for the trial on behalf of the Trial Sponsor and the Trial Funder and to ensure that the trial is conducted according to the guidelines for Good Clinical Practice (GCP), Research Governance Framework for Health and Social Care and all relevant regulations and local policies. The TSC will consider and act, as appropriate, upon the recommendations of the Data Monitoring Committee (DMC) and ultimately carries the responsibility for deciding whether a trial needs to be stopped on grounds of safety or efficacy.

The TSC will meet face-to-face or via teleconference at least once prior to recruitment of the first patient, then at least annually until the end of the DESTINY trial, and as required depending on the needs of the trial office.

Membership and duties/responsibilities are outlined in the TSC Charter. In summary, the TSC will: provide overall oversight of the trial, including the practical aspects of the study, as well as ensuring that the study is run in a way which is both safe for the participants and provides appropriate feasibility data to the sponsor and investigators.

14.5 Data Monitoring Committee

Data analyses will be supplied in confidence to an independent Data Monitoring Committee (DMC), which will be asked to give advice on whether the accumulated data from the trial, together with the results from other relevant research, justifies the continuing recruitment of further participants. The DMC will operate in accordance with a trial specific DMC Charter. The DMC will meet at least annually as agreed by the Committee and documented in the Charter. More frequent meetings may be required for a specific reason (e.g. safety phase) and will be recorded in minutes.

The DMC will be scheduled to meet prior to the recruitment of the first patient, in a joint meeting with the TSC, 6 months after the trial opens to recruitment and then every 6-12 months thereafter until the trial closes to recruitment.

Additional meetings may be called if recruitment is much faster than anticipated and the DMC may, at their discretion, request to meet more frequently or continue to meet following completion of recruitment. An emergency meeting may also be convened if a safety issue is identified. The DMC may consider recommending the discontinuation of the trial if the recruitment rate or data quality are unacceptable or if any issues are identified which may compromise participant safety. The trial would stop early if the interim analyses showed differences between treatments that were deemed to be convincing to the clinical community.

14.6 Finance

The British Heart Foundation (BHF) is funding this trial (CS/20/3/34738). It will be adopted to the National Institute for Health Research Clinical Research Network (CRN) portfolio and CRN support for the trial will be sought. Excess cost for the trial remains part of NHS costs while trial resources outside routine care and not covered by the CRN will be funded by the trial in the form of per patient payments to a maximum of £230 per patient.

15 ETHICAL CONSIDERATIONS

The trial will be conducted in accordance with the UK Policy Framework for Health and Social Care Research 2017, the applicable UK Statutory Instruments, which include the Medicines for Human Use Clinical Trials 2004 and subsequent amendments, the Data Protection Act 2018, and the Human Tissue Act 2004. This trial will be carried out under a Clinical Trial Authorisation in accordance with the Medicines for Human Use Clinical Trials regulations. The protocol will be submitted to and approved by the REC prior to circulation and the start of the trial. All correspondence with the MHRA and/or REC will be retained in the Trial Master File/Investigator Site File, and an annual progress report (APR) will be submitted to the REC within 30 days of the anniversary date on which the favourable opinion was given by the REC, and annually until the trial is declared ended.

Before any participants are enrolled into the trial, the PI at each site is required to obtain local R&D approval/assurance. Sites will not be permitted to enrol participants until written confirmation of R&D approval/assurance is received by the BCTU trials team.

It is the responsibility of the PI to ensure that all subsequent amendments gain the necessary local approval. This does not affect the individual clinicians' responsibility to take immediate action if thought necessary to protect the health and interest of individual participants.

16 CONFIDENTIALITY AND DATA PROTECTION

Personal data recorded on all documents will be regarded as strictly confidential and will be handled and stored in accordance with the provisions of the General Data Protection Regulation (GDPR) as implemented by the Data Protection Act 2018 (DPA 2018).

Participants will always be identified using their unique trial identification number on the Case Report Form and correspondence between the BCTU. Participants will give their explicit consent for the movement of their consent form, giving permission for BCTU to be sent a copy. This will be used to perform in-house monitoring of the consent process.

The Investigator must maintain documents not for submission to BCTU (e.g. Participant Identification Logs) in strict confidence. In the case of specific issues and/or queries from the regulatory authorities, it will be necessary to have access to the complete trial records, provided that participant confidentiality is protected.

BCTU will maintain the confidentiality of all participant's data and will not disclose information by which participants may be identified to any third party. Representatives of the DESTINY trial team and sponsor may be required to have access to participant's notes for quality assurance purposes, but participants should be reassured that their confidentiality will always be respected.

17 FINANCIAL AND OTHER COMPETING INTERESTS

The interventions used in the DESTINY trial are no longer protected by patent and are already in standard clinical use, del Nido in the United States and St. Thomas' in the United Kingdom. There are no commercial repercussions on using one intervention in preference to another. Members of the TSC and DMC are required to provide declarations on potential competing interests as part of their membership of the committees. Authors are similarly required to provide declarations at the time of submission to publishers.

18 INSURANCE AND INDEMNITY

The University of Birmingham has in place clinical trials indemnity coverage for this trial which provides cover to the University for harm which comes about through the University's, or its staff's, negligence in relation to the design or management of the trial and may alternatively, and at the University's discretion provide cover for non-negligent harm to participants.

With respect to the conduct of the trial at Site and other clinical care of the participant, responsibility for the care of the participants remains with the NHS organisation responsible for the Clinical Site and is therefore indemnified through the NHS Litigation Authority.

The University of Birmingham is independent of any pharmaceutical company, and as such it is not covered by the Association of the British Pharmaceutical Industry (ABPI) guidelines for participant compensation.

19 POST-TRIAL CARE

The clinical interventions used in the DESTINY trial are at a single point in time and cannot be amended in any way once performed. As such, there is no need to provide continuing post-trial care other than that used as standard local practice.

20 PUBLICATION POLICY

Results of this trial will be submitted for publication in a peer reviewed journal. The manuscript will be prepared by the Chief Investigator (Nigel Drury) and authorship will be determined by the trial publication policy to be agreed by the TMG.

Any secondary publications and presentations prepared by Investigators must be reviewed and approved by the TSC. Manuscripts must be submitted to the TSC in a timely fashion and in advance of being submitted for publication, to allow time for review and resolution of any outstanding issues. Authors must acknowledge that the trial was performed with the support of The University of Birmingham. Intellectual property rights will be addressed in the model Non-Commercial Agreement (mNCA) between Sponsor and site.

21 ACCESS TO FINAL DATA SET

The final dataset will be available to members of the Trial Management and co-applicant group who need access to the data to undertake the final analyses.

Requests for data generated during this study will be considered by BCTU. Data will typically be available within six months after the primary publication unless it is not possible to share the data (for example: the trial results are to be used as part of a regulatory submission, the release of the data is subject to the approval of a third party who withholds their consent, or BCTU is not the controller of the data).

Only scientifically sound proposals from appropriately qualified Research Groups will be considered for data sharing. The request will be reviewed by the BCTU Data Sharing Committee in discussion with the Chief Investigator and, where appropriate (or in absence of the Chief Investigator) any of the following: Trial Sponsor, relevant Trial Management Group (TMG), and independent Trial Steering Committee (TSC).

A formal Data Sharing Agreement (DSA) will be required between respective organisations once release of the data is approved and before data can be released. Data will be fully de-identified (anonymised) unless the DSA covers transfer of patient identifiable information. Any data transfer will use a secure and encrypted method.

22 REFERENCE LIST

1. Gaies MG, Gurney JG, Yen AH, Napoli ML, Gajarski RJ, Ohye RG, et al. Vasoactive-inotropic score as a predictor of morbidity and mortality in infants after cardiopulmonary bypass. *Pediatr Crit Care Med* 2010;11(2):234-8.
2. Gaies MG, Jeffries HE, Niebler RA, Pasquali SK, Donohue JE, Yu S, et al. Vasoactive-inotropic score is associated with outcome after infant cardiac surgery: an analysis from the Pediatric Cardiac Critical Care Consortium and Virtual PICU System Registries. *Pediatr Crit Care Med* 2014;15(6):529-37.
3. Brown KL, Pagel C, Brimmell R, Bull K, Davis P, Franklin RC, et al. Definition of important early morbidities related to paediatric cardiac surgery. *Cardiol Young* 2017;27(4):747-56.
4. Tibby SM, Murdoch IA. Monitoring cardiac function in intensive care. *Arch Dis Child* 2003;88(1):46-52.
5. Soler YA, Nieves-Plaza M, Prieto M et al. Pediatric Risk, Injury, Failure, Loss, End-Stage renal disease score identifies acute kidney injury and predicts mortality in critically ill children: a prospective study. *Pediatr Crit Care Med* 2013;14(4):e189-95.
6. National Institute for Cardiovascular Outcomes Research. Congenital Heart Disease. https://nicor4.nicor.org.uk/CHD/an_paeds.nsf/WMortality
7. Buckberg GD, Brazier JR, Nelson RL, Myron Goldstein S, McConnell DH, Cooper N. Studies of the effects of hypothermia on regional myocardial blood flow and metabolism during cardiopulmonary bypass: I. The adequately perfused beating, fibrillating, and arrested heart. *J Thorac Cardiovasc Surg* 1977;73(1):87-94.
8. Doenst T, Schlensak C, Beyersdorf F. Cardioplegia in pediatric cardiac surgery: do we believe in magic? *Ann Thorac Surg* 2003;75(5):1668-77.
9. Mildh LH, Pettilä V, Sairanen HI, Rautiainen PH. Cardiac troponin T levels for risk stratification in pediatric open heart surgery. *Ann Thorac Surg* 2006;82(5):1643-8.
10. Wittnich C, Peniston C, Ianuzzo D, Abel JG, Salerno TA. Relative vulnerability of neonatal and adult hearts to ischemic injury. *Circulation* 1987;76(5 Pt 2):V156-60.
11. Bolling K, Kronon M, Allen BS, Ramon S, Wang T, Hartz RS, et al. Myocardial protection in normal and hypoxically stressed neonatal hearts: the superiority of hypocalcemic versus normocalcemic blood cardioplegia. *J Thorac Cardiovasc Surg* 1996;112(5):1193-200; discussion 200-1.
12. Baker JE, Boerboom LE, Olinger GN. Age-related changes in the ability of hypothermia and cardioplegia to protect ischemic rabbit myocardium. *J Thorac Cardiovasc Surg* 1988;96(5):717-24.
13. Imura H, Caputo M, Parry A, Pawade A, Angelini GD, Suleiman MS. Age-dependent and hypoxia-related differences in myocardial protection during pediatric open heart surgery. *Circulation* 2001;103(11):1551-6.
14. del Nido PJ, Mickle DA, Wilson GJ, Benson LN, Weisel RD, Coles JG, et al. Inadequate myocardial protection with cold cardioplegic arrest during repair of tetralogy of Fallot. *J Thorac Cardiovasc Surg* 1988;95(2):223-9.

15. Harvey B, Shann KG, Fitzgerald D, Mejak B, Likosky DS, Puis L, et al. International pediatric perfusion practice: 2011 survey results. *J Extra Corpor Technol* 2012;44(4):186-93.
16. Kotani Y, Tweddell J, Gruber P, Pizarro C, Austin EH, 3rd, Woods RK, et al. Current cardioplegia practice in pediatric cardiac surgery: a North American multiinstitutional survey. *Ann Thorac Surg* 2013;96(3):923-9.
17. Reagor JA, Clingan S, Kulat BT, Matte GS, Voss J, Tweddell JS. The Norwood Stage 1 procedure - conduct of perfusion: 2017 Survey results from NPC-QIC member institutions. *Perfusion* 2018;33(8):667-78.
18. Drury NE, Horsburgh A, Bi R, Willetts RG, Jones TJ. Cardioplegia practice in paediatric cardiac surgery: a UK & Ireland survey. *Perfusion* 2019;34(2):125-9.
19. Ma M, Gauvreau K, Allan CK, Mayer JE, Jr., Jenkins KJ. Causes of death after congenital heart surgery. *Ann Thorac Surg* 2007;83(4):1438-45.
20. Gaies M, Pasquali SK, Donohue JE, Dimick JB, Limbach S, Burnham N, et al. Seminal Postoperative Complications and Mode of Death After Pediatric Cardiac Surgical Procedures. *Ann Thorac Surg* 2016;102(2):628-35.
21. Immer FF, Stocker F, Seiler AM, Pfammatter JP, Bachmann D, Printzen G, et al. Troponin-I for prediction of early postoperative course after pediatric cardiac surgery. *J Am Coll Cardiol* 1999;33(6):1719-23.
22. Drury NE, Yim I, Patel AJ, Oswald NK, Chong CR, Stickley J, et al. Cardioplegia in paediatric cardiac surgery: a systematic review of randomized controlled trials. *Interact Cardiovasc Thorac Surg* 2019;28(1):144-50.
23. Taggart DP, Hadjinikolas L, Hooper J, Albert J, Kemp M, Hue D, et al. Effects of age and ischemic times on biochemical evidence of myocardial injury after pediatric cardiac operations. *J Thorac Cardiovasc Surg* 1997;113(4):728-35.
24. Su JA, Kumar SR, Mahmoud H, Bowdish ME, Toubat O, Wood JC, et al. Postoperative Serum Troponin Trends in Infants Undergoing Cardiac Surgery. *Semin Thorac Cardiovasc Surg* 2019;31(2):244-51.
25. Talwar S, Bhoje A, Sreenivas V, Makhija N, Aarav S, Choudhary SK, et al. Comparison of del Nido and St Thomas Cardioplegia Solutions in Pediatric Patients: A Prospective Randomized Clinical Trial. *Semin Thorac Cardiovasc Surg* 2017;29(3):366-74.
26. Panigrahi D, Roychowdhury S, Guhabiswas R, Rupert E, Das M, Narayan P. Myocardial protection following del Nido cardioplegia in pediatric cardiac surgery. *Asian Cardiovasc Thorac Ann* 2018;26(4):267-72.
27. Negi SL, Mandal B, Singh RS, Puri GD. Myocardial protection and clinical outcomes in Tetralogy of Fallot patients undergoing intracardiac repair: a randomized study of two cardioplegic techniques. *Perfusion* 2019; 34: 495-502.
28. Rushel KZ, Hoque A, Alamgir MK, Islam MZ, Hasan KA, Rahman MR, et al. Comparative Study between the Use of Multidose Standard Cardioplegia and Long Acting Del Nido Cardioplegia during Intracardiac Repair for Tetralogy of Fallot in Pediatric Patients. *Mymensingh Med J* 2018;27(3):610-6.

29. 24. Gorjipour F, Dehaki MG, Totonchi Z, Hajimiresmaiel SJ, Azarfarin R, Pazoki-Toroudi H, et al. Inflammatory cytokine response and cardiac troponin I changes in cardiopulmonary bypass using two cardioplegia solutions; del Nido and modified St. Thomas': a randomized controlled trial. *Perfusion* 2017;32(5):394-402.

30. Buel ST, Striker CW, O'Brien JE. del Nido versus St. Thomas Cardioplegia Solutions: A Single-Center Retrospective Analysis of Post Cross-Clamp Defibrillation Rates. *J Extra Corpor Technol* 2016;48(2):67-70.

31. O'Brien JD, Howlett SE, Burton HJ, O'Blenes SB, Litz DS, Friesen CL. Pediatric cardioplegia strategy results in enhanced calcium metabolism and lower serum troponin T. *Ann Thorac Surg* 2009;87(5):1517-23.

32. Drury NE, Patel AJ, Oswald NK, Chong CR, Stickley J, Barron DJ, et al. Randomized controlled trials in children's heart surgery in the 21st century: a systematic review. *Eur J Cardiothorac Surg* 2018;53(4):724-31.

33. Matte GS, del Nido PJ. History and use of del Nido cardioplegia solution at Boston Children's Hospital. *J Extra Corpor Technol* 2012;44(3):98-103.

34. Powell WJ, Jr., DiBona DR, Flores J, Leaf A. The protective effect of hyperosmotic mannitol in myocardial ischemia and necrosis. *Circulation* 1976;54(4):603-15.

35. O'Blenes SB, Friesen CH, Ali A, Howlett S. Protecting the aged heart during cardiac surgery: the potential benefits of del Nido cardioplegia. *J Thorac Cardiovasc Surg* 2011;141(3):762-70.

36. van Emous JG, Nederhoff MG, Ruigrok TJ, van Echteld CJ. The role of the Na+ channel in the accumulation of intracellular Na+ during myocardial ischemia: consequences for post-ischemic recovery. *J Mol Cell Cardiol* 1997;29(1):85-96.

37. McGowan FX, Jr., Cao-Danh H, Takeuchi K, Davis PJ, del Nido PJ. Prolonged neonatal myocardial preservation with a highly buffered low-calcium solution. *J Thorac Cardiovasc Surg* 1994;108(4):772-9.

38. Takeuchi K, Cao-Danh H, Kawai A, Ohkado A, Konishi H, McGowan FX, et al. Prolonged preservation of the blood-perfused canine heart with glycolysis-promoting solution. *Ann Thorac Surg* 1999;68(3):903-7.

39. Drury NE, Najdekr L, Lee JJ, Guo N, Madhani M, Dunn WB. The effects of three cardioplegia solutions on myocardial metabolism during ischaemia-reperfusion in a Langendorff mouse model. Submitted for publication.

40. Pourmoghadam KK, Ruzmetov M, O'Brien MC, Piggott KD, Plancher G, Narasimhulu SS, et al. Comparing del Nido and Conventional Cardioplegia in Infants and Neonates in Congenital Heart Surgery. *Ann Thorac Surg* 2017;103(5):1550-6.

41. Doenst T, Borger MA, Weisel RD, Yau TM, Maganti M, Rao V. Relation between aortic cross-clamp time and mortality--not as straightforward as expected. *Eur J Cardiothorac Surg* 2008;33(4):660-5.

42. Mishra P, Jadhav RB, Mohapatra CKR, Khandekar J, Raut C, Ammannaya GK, et al. Comparison of del Nido cardioplegia and St. Thomas Hospital solution - two types of cardioplegia in adult cardiac surgery. *Kardiochir Torakochirurgia Pol* 2016;13(4):295-9.

43. NHS Data Model and Dictionary. Paediatric Critical Care Minimum Data Set. https://www.datadictionary.nhs.uk/data_dictionary/messages/supporting_data_sets/data_sets/paediatric_critical_care_minimum_data_set_fr.asp?shownav=1

44. Virzi L, Pemberton V, Ohye RG et al. Reporting adverse events in a surgical trial for complex congenital heart disease: the Pediatric Heart Network experience. *J Thorac Cardiovasc Surg* 2011 Sep;142(3):531-7.

45. Healthcare Quality Improvement Partnership. National Congenital Heart Disease Audit (NCHDA): 2020 Summary Report. London: NICOR, 2020.

46. Ohye RG, Sleeper LA, Mahony L, Newburger JW, Pearson GD, Lu M et al. Comparison of shunt type in the Norwood procedure for single ventricle lesions. *N Engl J Med* 2010;362:1980-92.

47. Squires RH Jr, Shneider BL, Bucuvalas J. Acute liver failure in children: the first 348 patients in the pediatric acute liver failure study group. *J Pediatr* 2006;148:652-658.

48. Leteurtre S, Martinot A, Duhamel A, Proulx F, Grandbastien B, Cotting J et al. Validation of the paediatric logistic organ dysfunction (PELOD) score: prospective, observational, multicentre study. *Lancet* 2003;362(9379):192-7.
