# Supplementary material for: del Nido versus St. Thomas’ blood cardioplegia in the young (DESTINY) trial: protocol for a multicentre randomised controlled trial in children undergoing cardiac surgery
Source: BMJ Open. 2025 Apr 14;15(4):e102029. doi: 10.1136/bmjopen-2025-102029 (PMC11997810; doi:10.1136/bmjopen-2025-102029)
Supplement: online supplemental file 2 [file bmjopen-15-4-s002.pdf]

<insert Trust logo>

PARENT / GUARDIAN INFORMATION SHEET

del Nido versus St Thomas' blood cardioplegia in the young (DESTINY) trial: a multi-centre randomised controlled trial in children undergoing cardiac surgery

Principal Investigator: <insert name>, <insert PI role >

An invitation to participate in research: The Heart Surgery team at <insert name> Hospital would like to invite your child to take part in a research study. Before you decide, you need to understand why the research is being done and what it would involve for you and your child. Please read the following information carefully and take time to decide whether or not you would like your child to take part. If there is anything that is not clear or you would like more information, please ask.

Trial Summary

- Children with congenital heart defects often need operations to correct the abnormalities that they were born with.
- The surgery is complex and usually involves a period of support on a heart-lung machine (cardiopulmonary bypass). This allows the heart to be stopped for a short time, using a fluid called *cardioplegia*, to complete the repair.
- This study compares two types of cardioplegia to see which one is better for children's recovery: St Thomas' is the most commonly used in the UK whilst del Nido is the standard of care in the US. With your consent, your child will be randomly assigned to receive either one of these types of cardioplegia.
- After surgery blood samples will be taken from your child to detect any injury to the heart; in total, this would be approximately 12ml (2-3 teaspoons) of blood and taking these samples will have no impact on their recovery.
- After surgery, your child will be discharged home and kept under regular follow-up in clinic. You will not need to attend any additional clinic visits for the study.
- This study will increase our understanding of which cardioplegia is better in children but there may not be any direct benefit for your child.
- Trial participation and information provided will be kept confidential and handled in accordance with Data Protection Act 2018.
- The rest of this Information Sheet provides more details about the trial and if you have any questions, please ask a member of the research team.

Why is my child being invited to take part?

Your child has been referred to the Heart Surgery team for an operation to repair their heart defect. We are performing a clinical trial in children undergoing heart surgery and have approached you to see if you would like your child to take part.

What is the aim of the study?

This study aims to improve the outcomes of children's heart surgery so that they recover faster with fewer complications. Children with congenital heart defects often need operations to correct the abnormalities that they were born with. The surgery is complex and usually involves a period of support on a heart-lung machine (cardiopulmonary bypass). This allows the heart to be stopped for a short period of time, using a fluid called *cardioplegia solution*, whilst the defect is repaired. Inevitably, any surgery puts a strain on the heart and has the potential to cause damage. In this study, we wish to compare two types of cardioplegia solution used to stop the heart: del Nido, the most commonly used in children in the US, and St Thomas', currently the standard practice in the UK, to determine whether del Nido protects the heart better than St Thomas', and whether children recover faster and with fewer complications, so that we can improve the outcomes of children's heart surgery.

Why do you need to stop the heart using cardioplegia?

During the operation, the heart will need to be stopped for a period of time so that the surgeon can repair the heart defect. Being involved in this study will <u>not</u> affect whether the heart needs to be stopped during the operation – the only change will be the type of cardioplegia solution that is used. Both del Nido and St Thomas' cardioplegia solutions are in routine clinical use in hospitals around the world and have been used in many thousands of children's heart operations.

Why might one cardioplegia solution be better than another?

Both del Nido and St Thomas' solutions are mixed with your child's blood as they are given through the heart-lung machine, and both stop the heart. However, the two solutions work in slightly different ways – del Nido cardioplegia requires a lower dose than St Thomas' to keep the heart still for longer but we do not know whether this also reduces any damage to the heart. So we are performing this study to see which solution is better at protecting the heart, leading to a quicker recovery, in children of different ages undergoing surgery for congenital heart disease in the UK.

Which cardioplegia solution will my child receive?

This study is a blinded, randomised controlled trial. This means that if you agree for your child to take part, they will be randomly allocated by computer with a 50% chance of receiving del Nido cardioplegia and a 50% chance of receiving St Thomas'. All other aspects of the anaesthetic, surgery and post-operative care will be the same and *neither* you *nor* the intensive care team will know which cardioplegia solution your child received. The theatre team will know as the two solutions are given in slightly different ways but will not be able to tell you. At the end of the study, the code will be revealed to see which children were in which group. This is a standard technique for preventing the doctors and nurses involved in a clinical trial from potentially influencing the results.

What will happen if I agree for my child to take part?

In addition to the standard operation and post-operative care, if you agree for your child to take part, you will be asked to sign a consent form and the following will occur:

- Your child's Paediatric Cardiologist and with your permission, your child's GP will be informed of their participation.
- Your child will be allocated to either the del Nido or St Thomas' group at random.
- Prior to surgery, all children have small plastic tubes (lines) inserted into their blood vessels to make measurements and take blood samples. In addition to routine blood samples, children in the study will have additional samples taken from these lines (no extra needles) over the first 24 hours after surgery to detect any injury to the heart; in total, this would be about 12ml (2-3 teaspoons) of blood and taking these samples will have no impact on their recovery.
- At the time of surgery, with your permission, we will also take a blood sample for genetic testing to see if there is a link between genetics and how well the heart recovers following surgery; this would be about 6ml (just over a teaspoon) of blood. These tests will be separate to any genetic tests that may be required by your child's clinical team and are extremely unlikely to have any implications for you or your child. The results will <u>not</u> be shared with any external bodies, including insurance companies. This part of the study is optional so if you do not wish for this sample to be taken, your child can still take part in the rest of the study.
- During surgery, to repair the defect, the heart is opened through a cut in the side of the right atrium, one of the upper chambers of the heart. In the study, two small biopsies of the atrium will be taken from the edge of the cut by the surgeon. Once the

heart defect is repaired, the cut will be closed in the normal way; taking the biopsies does not increase the risk of the operation in any way but will allow us to understand how the different cardioplegia solutions work to protect the heart. This part of the study is optional so if you do not wish for these samples to be taken, your child can still take part in the rest of the study.

- In some children, bands of muscle inside the heart which block the flow of blood to the lungs are routinely removed as part of the operation; if your child is in the study, the removed muscle may be kept for analysis rather than be thrown away.
- After surgery, your child will be discharged home and kept under regular follow-up in the clinic; you will not need to attend any additional clinic visits for the study.

What are the benefits?

This trial will increase our understanding of which cardioplegia is better in children but there may not be any direct benefit for your child. Whilst some previous studies have suggested that del Nido cardioplegia may better protect children's hearts during surgery, we do not know if it is beneficial to all children and whether they recover faster with fewer complications - that is why we are conducting this study. We do not know whether being in the study will make your child's surgery safer, but we are conducting it to understand how to improve the outcomes of children's heart surgery in the future.

Are there any risks?

Both types of cardioplegia are used routinely for heart surgery in children, del Nido in the US and St Thomas' in the UK. The operation itself carries a risk, as discussed with you by your Surgeon and Cardiologist, but being involved in this study causes no additional pain, discomfort, distress or intrusion. If your child does not take part in this study, the surgeon will still need to use a cardioplegia solution to perform the operation.

How many children will be taking part in this study?

We aim to recruit 220 children undergoing surgery for congenital heart defects at several UK heart surgery centres to take part in this study.

Does my child have to take part?

Taking part in the study is entirely voluntary – you decide. This parent information sheet gives you information about the study and we can answer any questions that you may have after reading it. Before your child's surgery, one of the research team will ask you whether you wish your child to participate in this study and if so, to sign a consent form.

Your child will only be included in the study if you give your express permission. Indeed, you are free to withdraw your child at any time, without giving a reason – their surgery will proceed as planned using standard cardioplegia, without any additional measurements or tests, and it will not affect the standard of their care. Neither you nor your child will receive any payment for taking part in this trial.

What happens to my child's information?

All necessary measures will be taken to keep your child's data safe and to comply with the provisions of the General Data Protection Regulation (GDPR) and Data Protection Act 2018.

How will we use information about your child?

We will need to use information from your child's medical records, including:

- Your child's name
- Your child's NHS number

All information collected on children who participate in this study will be securely stored on Hospital and University computers and will only be accessible by authorised personnel. The only people in the University of Birmingham who will have access to information that identifies your child will be people who manage the study or audit the data collection process. Paper copies of the data will be stored in a locked office at the Hospital and with your permission, a copy of your signed consent form will be sent to the trial office. All data will be coded and kept confidential, ensuring that your child's identity will not be revealed without your express permission. In the trial office, your child will be identified by a unique study number and in routine communication with the hospital, your child will only be identified by their study number, initials, and date of birth; data may be provided to the trial office on paper or electronically.

What are my choices about how my child's information is used?

The trial team will use this data to conduct and analyse the study and it may be necessary to allow authorised personnel from government regulatory agencies, the sponsor and NHS bodies to have access to your child's medical and research records, to ensure that the study is being conducted to the highest standards. Anonymised data from the study may be provided to other third parties for research, safety monitoring or licensing purposes but will only be granted if the necessary procedures and approvals are in place. This information will not identify your child and will not be combined with other information

in a way that could identify your child. It will only be used for the purpose of health research and cannot be used to contact you or to affect your child's care. It will not be used to make decisions about future services available to your child, such as insurance. Under no circumstances will your child be identified in any way in any report, presentation or publication arising from this or any other study. Your rights to access, change or move your child's data are limited, as we need to manage their information in specific ways for the research to be reliable and accurate. If you withdraw your child from the study, we will keep the information about them that we have already obtained; to safeguard their rights, we will use the minimum personally identifiable data possible. Anonymised data from the study will be analysed, presented at scientific meetings and published in medical journals to inform other doctors and health professionals of the research findings.

At the end of the study, we will also make the anonymised data publicly available so that other researchers have the opportunity to analyse it and answer other important questions about heart surgery and cardioplegia. This information will not identify your child and will not be combined with other information in a way that could identify your child. The information will only be used for the purpose of health research and could not be used to contact you or to affect your child's care. It will not be used to make decisions about future services available to your child, such as insurance. Under no circumstances will your child be identified in any way in any report, presentation or publication arising from this or any other study. Following completion of the study, the data will be kept for 25 years then destroyed in accordance with national guidance.

Where can I find out more about how my child's information is used?

As sponsor of this study, the University of Birmingham is the data controller, responsible for protecting your child's data; for more information, please contact the University's Data Protection Officer: dataprotection@contacts.bham.ac.uk or the local research team. You can also visit www.hra.nhs.uk/information-about-patients/ or download the leaflet available from www.hra.nhs.uk/patientdataandresearch.

What happens to my child's samples?

All blood and tissue samples collected during the study will be stored in secure laboratories at the hospital, collaborating organisations and the University in accordance with the Human Tissue Act 2004. Once analysed, any remaining samples may be kept and used in future research studies in the UK which conform to all relevant legal, governance and ethical requirements.

What will happen if new information becomes available during the study?

Sometimes during a study, new information about the intervention being studied becomes available. If this happens, a member of the research team will tell you and discuss whether your child should continue in the study. You will have the option to decide whether you wish them to continue or not and a member of the research team may ask you to re-sign a consent form if you decide you wish them to continue.

What if something goes wrong?

The standard care of children undergoing heart surgery involves intensive monitoring and we do not expect the study itself to cause any problems. Complications of surgery can occur and these will be dealt with in the normal manner, regardless of the research study. Your child's safety during and after surgery is paramount. In the very unlikely event that any harm should occur because of taking part in this study, we want you to be informed of your rights. There are no special compensation arrangements, but you may have the right to claim damages in a court of law; this would require you to prove fault on the part of the NHS Trust, University or any manufacturer involved. The standard NHS complaints mechanisms are available to you; further information can be obtained from the Patient Advice & Liaison Service (PALS) at <insert name> Hospital on <insert number>. If you wish to complain about how we have handled your child's personal data, you can contact our Data Protection Officer who will investigate the matter: <insert name>, <insert contact>. If you are not satisfied with our response or believe we are processing your child's personal data in a way that is not lawful you can complain to the Information Commissioner's Office (ICO).

What happens at the end of the study?

At the end of the study, your child's treatment and follow-up continues as would that of a child who had not been involved in the study. However, we would like to monitor your child's health status by accessing data held centrally by NHS Digital and other NHS organisations; with your permission, we will continue to hold your child's name, date of birth, postcode, and NHS number, which may be shared with these central NHS bodies. With your permission, we may also contact you in the future for a follow-up study to look at the long-term effects of surgery during childhood on the heart.

Will I ever know if the trial worked and which treatment my child received?

Yes – but not until the whole study has finished and we have analysed the results. We will work with the *Children's Heart Federation*, the UK's leading national children's heart charity, to produce a newsletter with the findings of the study to send to the parents of all children involved. At this stage, we will be able to tell you which group your child was in and so which cardioplegia solution they had received.

Who is organising, funding, and insuring this research?

This study has been organised and developed by the teams at Birmingham Children's Hospital, Bristol Royal Hospital for Children, Great Ormond Street Hospital for Children, Leeds Children's Hospital and Birmingham Clinical Trials Unit. It is funded by the **British Heart Foundation** and sponsored by the University of Birmingham, who has obtained Clinical Trials insurance cover for this trial from UM Association Limited.

Have parents been involved in developing this study?

Yes – the parents of children who have previously undergone cardiac surgery were invited to take part in a focus group to explore their thoughts on participation in a trial of cardioplegia. They would have been keen for their child to participate if: it may benefit their child or others in the future; the solution is already proven and in use elsewhere; it is not going to cause harm; and their surgeon thinks it is a good idea. They were also in favour of comparing the 'standard treatments' used in the UK and the US.

Who has reviewed this research study and leaflet?

The study has been reviewed by the British Heart Foundation, the NHS Research & Development teams at the participating hospitals and the University of Birmingham. It has been given a favourable opinion for conduct in the NHS by the West Midlands-Solihull Research Ethics Committee. The child and parent information sheets have been reviewed and revised by children who have had heart surgery in Bristol and their parents.

Questions? Contact the DESTINY Trial Office at the Birmingham Clinical Trials Unit, University of Birmingham, Edgbaston, Birmingham B15 2TT, tel: 0121 415 8444, email: destiny@trials.bham.ac.uk, or your local research team: <insert name>, Lead Research Nurse at <insert name>, tel: <insert number>, email: <insert email>.

Flow chart for the del Nido v St Thomas' blood cardioplegia in the young trial

You will have been given this information sheet by your Consultant or a member of the research team in the clinic or been sent it in the post.

↓

Please read it carefully and consider whether you would like your child to take part. If you have any questions, please feel free to ask.

↓

We will contact you either by telephone, in the pre-operative assessment clinic or when your child is admitted for their operation.

↓

If you are happy for your child to take part, you will be asked to sign a Consent form by a member of the research team.

↓

On the day of surgery, you may go along to theatre with your child as usual. The computer will allocate them to a group and they will receive *either* del Nido *or* St Thomas' cardioplegia during their operation. During surgery, blood +/- tissue samples will be removed for analysis.

↓

After the operation, they will be transferred to the Paediatric Intensive Care Unit (PICU) as usual and you will be able to see them there.

↓

Whilst they are recovering on PICU, several additional blood tests will be performed using the lines already in place – no new needles required.

↓

When they are well enough, your child will be transferred to the ward and then discharged from hospital once they are ready to go home.

↓

After discharge, they will be seen regularly in the outpatient clinic but there will be no additional follow-up appointment related to the study.

↓

Once the trial has completed, we will send you a newsletter with the results.

Thank you for reading this information & considering your child's participation
