# Supplementary material for: del Nido versus St. Thomas’ blood cardioplegia in the young (DESTINY) trial: protocol for a multicentre randomised controlled trial in children undergoing cardiac surgery
Source: BMJ Open. 2025 Apr 14;15(4):e102029. doi: 10.1136/bmjopen-2025-102029 (PMC11997810; doi:10.1136/bmjopen-2025-102029)
Supplement: online supplemental file 4 [file bmjopen-15-4-s004.pdf]

<insert Trust logo>

CHILD / YOUNG PERSON INFORMATION SHEET

del Nido versus St Thomas' blood cardioplegia in the young

Principal Investigator: <insert name>, <insert PI role>

For child/young person aged 8 years and above, if appropriate, with parent/guardian

The Heart Surgery team at <insert hospital> Hospital would like to invite you to take part in our research study. Please read this information sheet carefully, discuss it with your parents or others, and decide whether you would like to take part. If there is anything that is not clear or you would like more information, please ask your doctor or nurse.

Why am I being invited to take part?

You have a heart condition and have been referred to the Heart Surgery team for an operation to repair it.

What is the aim of the study?

During the operation, we use a type of fluid called cardioplegia to protect your heart from being damaged. In this study, we are trying to find out whether one type of cardioplegia is better at protecting children's hearts than another type. The types of cardioplegia that we are looking at are called *del Nido* and *St Thomas'* and they are both used routinely during children's heart operations around the world. We want to see which one will help children like you recover faster and more easily after surgery.

What will happen if I take part in this study?

If you take part, we will use one of these two types of fluid to protect your heart during the operation. The doctors doing your operation will know which type they are using but they will not tell you, your parents or the other doctors and nurses looking after you and will look after you exactly in the same way. After your operation, we will take some blood tests, but you will not need any extra needles as these will be taken through small plastic tubes put in just before the operation. When you are better, you will go home as planned.

Will taking part in this study help me?

We do not know if one cardioplegia is better than the other and that is what you will help us to find out. When we know that, we will be able to use that information to better help children having heart surgery in the future.

Do I have to take part?

You do not need to take part in this study if you do not want to – it is up to you. If you decide that you do not want to take part in the study, we will still look after you and you will still get exactly the same treatment.

What if I don’t want to be in the study anymore?

If you decide in the future that you do not want to take part in the study anymore, just tell your parents, doctors, or nurses. Your operation will continue, and we will use standard cardioplegia without any study measurements.

How many other children will be taking part in this study?

We are asking 220 children having heart operations at four hospitals to take part.

What happens at the end of the study?

At the end of the study, your treatment and follow-up will continue as normal – you will not have to come back to hospital or have any additional tests. We will keep a check on how well you are doing by looking at what your doctors say about you.

Will I ever know the results of the study and which treatment I had?

Yes – when we have finished the study, we will write to you and your parents with the results and let you know which type of cardioplegia you had during your operation.

Has anyone checked that this study is ok to do?

Before any study goes ahead, it is checked by a group of people called an ethics committee to make sure that it is safe and fair. This study has also been checked by the British Heart Foundation who are paying for the study.

What happens now? If you are happy to take part, your mum or dad will be asked to sign a form to say that they also agree for you to take part.

Thank you for reading this information and considering whether to take part
