# Supplementary material for: del Nido versus St. Thomas’ blood cardioplegia in the young (DESTINY) trial: protocol for a multicentre randomised controlled trial in children undergoing cardiac surgery
Source: BMJ Open. 2025 Apr 14;15(4):e102029. doi: 10.1136/bmjopen-2025-102029 (PMC11997810; doi:10.1136/bmjopen-2025-102029)
Supplement: online supplemental file 6 [file bmjopen-15-4-s006.pdf]

<insert Trust logo>

PARENT / GUARDIAN CONSENT FORM

del Nido versus St Thomas' blood cardioplegia in the young (DESTINY) trial: a multi-centre randomised controlled trial in children undergoing cardiac surgery

Principal Investigator: <insert name>, <insert PI role>

Please initial boxes

1. I confirm that I have read and understand the parent/guardian information sheet (version .... dated ....) for the above study. I have had the opportunity to consider the study information, ask questions and have had these answered satisfactorily. ☐

2. I understand that my child's participation is voluntary and that I am free to withdraw at any time, without giving a reason and without my child's care or legal rights being affected. I understand that data collected up to my time of withdrawal may still be used. ☐

3. I understand that relevant sections of my child's medical notes and data collected during the study may be looked at by responsible individuals from the NHS Trusts, the University of Birmingham, or the regulatory authorities, where it is relevant to my taking part in this research. I give permission for these individuals to have access to my child's records. ☐

4. I consent to the storage, including electronic, of personal information for the purposes of this study. I understand that any information that could identify me or my child will be kept strictly confidential, and that no personal information will be included in the study report or any other publication. ☐

5. Data collected that identifies my child by name, such as this consent form, will be transferred from the hospital to be stored during the trial at the University of Birmingham and after the trial at a specialist archiving facility, in compliance with current regulations. I agree to the transfer and storage of this data. ☐

6. I understand that my child's data collected during this study may be shared with academic collaborative third parties who will help with the analysis of this and future studies. Before sharing, any information that could identify me or my child (e.g. name, date of birth, contact details) will be removed and replaced with a trial number. ☐

7. I understand that blood samples will be kept for the purposes of research and I give permission for these samples to be taken and stored. I agree to the transfer of these samples to collaborating organisations for analysis. ☐

Please initial boxes

8. I agree to my child's GP and/or other doctors involved in their care, being informed of my child's participation in the study. ☐

9. I agree to my child's participation in the study. ☐

Optional:

A. *Blood samples*: I understand that any remaining blood samples may be stored beyond the end of this trial and used in future research which conforms to all relevant legal, governance and ethical requirements. ☐

B. N/A

C. *Genetics*: I understand that a sample will be kept for research aimed at understanding the genetic influences on disease and recovery from surgery and I give permission for this sample to be taken and stored. I agree to the transfer of these samples to collaborating organisations for analysis. I understand that the results of these tests are very unlikely to have any implications for me or my child. ☐

D. *Long-term health status*: I agree to the information held and maintained by the Health and Social Care Information Centre, NHS Digital, and other current & future NHS bodies, being used in the future to provide information about my child's long-term health status and healthcare. For this purpose, I agree to Birmingham Clinical Trials Unit holding my child's name, date of birth, postcode, and NHS number, which may be shared with these central NHS bodies. ☐

E. *Future contact*: I consent to the study team retaining contact information for my child and I so that they may contact me in the future about my child's health status and provide information so that I may consider my child's participation in one or more follow-up studies. ☐

Name of Child: Date of birth:

______________________	______________________	____________
Name of Parent/Guardian	Signature of Parent/Guardian	Date signed
______________________	______________________	____________
Name of Investigator	Signature of Investigator	Date signed
