# Supplementary material for: del Nido versus St. Thomas’ blood cardioplegia in the young (DESTINY) trial: protocol for a multicentre randomised controlled trial in children undergoing cardiac surgery
Source: BMJ Open. 2025 Apr 14;15(4):e102029. doi: 10.1136/bmjopen-2025-102029 (PMC11997810; doi:10.1136/bmjopen-2025-102029)
Supplement: online supplemental file 7 [file bmjopen-15-4-s007.pdf]

British Heart Foundation DESTINY UNIVERSITY OF BIRMINGHAM BCTU Birmingham Clinical Trials Unit <insert Trust logo>

CHILD / YOUNG PERSON ASSENT FORM

del Nido versus St Thomas' blood cardioplegia in the young

Principal Investigator: <insert name>, <insert PI role>

To be completed by child aged 8 years and above, if appropriate, with parent/guardian

Please *circle* all of the answers that you agree with:

Do you understand what this study is about?	Yes	No
Have you asked all of the questions you want?	Yes	No
Have you had your questions answered in a way you understand?	Yes	No
Are you happy to take part?	Yes	No

If any answers are 'No' or you don't want to take part, don't write your name below.

If all answers are 'Yes' and you do want to take part, please write your name below:

Your name: ___________________________________

Date: ____

The person who explained this study to you needs to sign too:

___________________________ ___________________________ _____________

Name of Investigator Signature of Investigator Date signed

Once completed: 1 for child/parent(s), 1 for medical notes and 1 (original) for trial site file
