# Supplementary material for: del Nido versus St. Thomas’ blood cardioplegia in the young (DESTINY) trial: protocol for a multicentre randomised controlled trial in children undergoing cardiac surgery
Source: BMJ Open. 2025 Apr 14;15(4):e102029. doi: 10.1136/bmjopen-2025-102029 (PMC11997810; doi:10.1136/bmjopen-2025-102029)
Supplement: online supplemental file 8 [file bmjopen-15-4-s008.pdf]

Supplemental table S1. Schedule of assessments: screening, consent, randomisation, intervention, outcome data, blood, and tissue samples.

	Pre-operative	Pre-sternotomy	Intraoperative			Before leaving theatre	On ICU admission	Time since aortic cross-clamp removal (hours)						Daily until discharge	Hospital discharge
			Onset ischaemia	During ischaemia	Late ischaemia			3	6	9	12	24	48		
Screening	x														
Consent	x														
Randomisation	x														
Clinical baseline data	x														
Blood for hs-troponin-I		x						x	x	x	x	x			
Arterial and central venous blood gases		x						x	x	x	x				
Administer IMP or control cardioplegia			x												
Intraoperative biopsies			x	x	x										
Conceal perfusion chart						x									
Inotrope data							x	x	x	x	x	x	x		
Other clinical outcome data							x				x	x	x	x	x
SAE reporting if required												x	x	x	x

IMP, investigational medicinal product; ICU, intensive care unit; SAE, serious adverse event.
